# Prediction of Hole Expansion Ratio in Advanced High-Strength Steels Using Physics-Informed Machine Learning

**DOI:** 10.3390/ma19081592

**Published:** 2026-04-15

**Authors:** Saurabh Tiwari, Khushbu Dash, Seongjun Heo, Nokeun Park, Nagireddy Gari Subba Reddy

**Affiliations:** 1 School of Materials Science and Engineering, Yeungnam University, Gyeongsan 38541, Republic of Korea; saurabht@yu.ac.kr (S.T.); transballer@gmail.com (S.H.); 2 Department of Chemical Engineering, Amrita Vishwa Vidyapeetham, Chennai Campus, Chennai 601103, India; d_khushbu@ch.amrita.edu; 3 Institute of Materials Technology, Yeungnam University, Gyeongsan 38541, Republic of Korea; 4 Department of New Materials Engineering, Engineering Research Institute, Gyeongsang National University, Jinju 52828, Republic of Korea

**Keywords:** hole expansion ratio, advanced high-strength steel, machine learning, synthetic data, gradient boosting, formability prediction

## Abstract

The hole expansion ratio (HER) is a critical formability metric for advanced high-strength steels (AHSS) in automotive applications; however, its experimental determination is costly and time-consuming. This study presents a machine learning framework for HER prediction using physics-informed synthetic data generation to address data scarcity challenges. A dataset of 300 AHSS conditions was generated based on validated empirical relationships from the literature, incorporating chemical composition, microstructure fractions, and mechanical properties. Multiple machine learning algorithms were evaluated, with the optimized Gradient Boosting model achieving excellent predictive performance on an independent test set (R^2^ = 0.80, RMSE = 5.81%, MAE = 4.93%). The feature importance analysis revealed physically meaningful rankings, with the ultimate tensile strength dominating (40.9%), followed by the bainite volume fraction (15.1%), martensite volume fraction (14.7%), and strain hardening exponent (12.4%). These rankings align with the established metallurgical understanding, thereby validating our synthetic data approach. The results demonstrate that machine learning models trained on physics-informed synthetic data can accurately predict the HER values with errors comparable to the experimental variability, providing a practical tool for accelerated AHSS design and optimization in automotive applications.

## 1. Introduction

Advanced high-strength steels (AHSS) have become indispensable materials in the automotive industry, driven by the simultaneous demands for improved fuel efficiency, enhanced crashworthiness, and reduced vehicle weight [1,2]. Among the AHSS grades, dual-phase (DP) steels have achieved particular commercial success owing to their favorable combination of high strength and adequate ductility [3]. The microstructure of DP steels consists of a soft ferrite matrix containing dispersed martensite islands, which is achieved through controlled intercritical annealing, followed by rapid cooling [4]. This unique microstructural architecture provides a low yield strength relative to the ultimate tensile strength, continuous yielding behavior, high work-hardening rates, and good uniform elongation, making DP steels especially suitable for complex stamping operations in automotive body structures [5]. However, the application of AHSS in automotive manufacturing faces persistent challenges related to its edge formability. The hole expansion test, standardized as ISO 16630, is the primary method for assessing stretch flangeability and edge-cracking resistance [6,7]. This test quantifies a material’s capacity to undergo localized stretching around a sheared or punched hole before fracture initiation at its edge. The hole expansion ratio is mathematically defined as HER (%) = [(df − d0)/d0] × 100, where d0 represents the initial hole diameter and df represents the final hole diameter at which through-thickness cracking first appears [8]. The punching or shearing process creates a work-hardened, damaged edge zone containing microvoids and microcracks that serve as preferential sites for crack initiation during subsequent forming operations [9,10]. This edge damage becomes increasingly severe in high-strength materials, where the punching forces are elevated and the local strain concentrations are intense [11,12].

Controlling and predicting edge formability remains substantially difficult because of the multiple interacting factors. First, the edge quality exhibits large variations depending on the punch clearance, punch sharpness, die condition, and cutting speed [13]. Second, die wear accelerates dramatically when processing ultra-high-strength grades, leading to the progressive degradation of the edge quality during production runs. Third, microstructural heterogeneity at the mesoscale creates localized weak paths where cracks propagate preferentially [14]. Fourth, the complex stress state during hole expansion involves through-thickness strain gradients, biaxial tension, and bending components, which challenge simple analytical prediction methods [15].

Experimental hole expansion testing requires specialized equipment, skilled operators for consistent crack detection, and careful specimen preparation to ensure reproducibility [16]. A typical automotive development program exploring multiple steel grades and heat treatment conditions may require dozens to hundreds of hole expansion tests, representing a substantial investment in time, materials, and labor costs. Recent advances in machine learning have demonstrated the potential of predicting the mechanical properties of metallic materials from composition and microstructure data, potentially reducing the experimental testing burden [17,18,19]. In the specific context of HER prediction, Kim et al. [20] applied multiple machine learning algorithms, including support vector machines, symbolic regression, extreme gradient boosting, and extra tree regressors, to a dataset of experimental steel conditions. Their analysis revealed that the bainite volume fraction was the most significant promoting factor for the HER, whereas the martensite volume fraction and carbon equivalent content were critical depressing factors. The machine learning models achieved R^2^ values exceeding 0.85, demonstrating that complex composition-microstructure-property relationships can be captured using data-driven approaches. Park et al. [21] developed an integrated machine vision and deep learning system for automated HER measurement, addressing the challenge of subjective crack detection. Additional studies have explored the correlations between tensile properties and hole expansion behavior using various regression techniques [22,23].

Despite these encouraging results, a fundamental obstacle impedes the broader application of machine learning to formability prediction: the scarcity of comprehensive and publicly available experimental datasets. High-quality mechanical property data remain largely proprietary, generated at substantial expense by steel producers and automotive manufacturers, who treat such data as competitive assets [24]. Academic studies typically focus on narrow compositional ranges or specific processing routes, generating datasets that are statistically insufficient for training robust machine-learning models [25]. The resulting data scarcity creates a challenging situation in which machine learning methods that require large datasets to reach their full potential cannot be developed or validated properly. This chicken-and-egg problem has motivated researchers to explore alternative data generation strategies. Synthetic data generation has emerged as a powerful approach for addressing data scarcity in diverse machine-learning applications [26,27]. In materials science, specifically in physics-informed synthetic data generation, where artificial data are created based on validated empirical relationships and fundamental physical models, has shown particular promise [28,29]. Unlike purely random data generation, which may create physically impossible material combinations, physics-informed approaches encode domain knowledge directly into data generation algorithms, ensuring that the resulting synthetic samples obey the known constraints and correlations [30]. Successful applications in materials informatics have demonstrated that carefully constructed synthetic datasets can serve as effective training data when the generation methodology is transparent, the underlying physical relationships are well validated, and the learned models are verified against real experimental measurements, where available [31,32]. The present study addresses the data scarcity challenge for hole expansion ratio prediction by developing a physics-informed synthetic data generation framework based on validated empirical relationships from the peer-reviewed literature. The specific objectives were as follows: (1) to generate a synthetic dataset of 300 AHSS conditions incorporating the composition, microstructure, and mechanical properties based on published correlations; (2) to evaluate the predictive performance of multiple machine learning algorithms, including linear models, tree-based ensembles, and support vector methods; (3) to perform systematic hyperparameter optimization to identify the best-performing model configuration; (4) to analyze feature importance rankings to validate physical consistency with metallurgical principles; and (5) to assess model generalization through a rigorous train-validation-test methodology with independent hold-out data. This approach provides a practical pathway for accelerated AHSS design while maintaining physical fidelity and enabling future validation of the results against experimental measurements.

## 2. Materials and Methods

### 2.1. Physics-Informed Synthetic Data Generation

The foundation of this study rests on the generation of synthetic data that respects the established physical relationships between composition, microstructure, mechanical properties, and hole expansion behavior. Rather than creating arbitrary data points, the generation algorithm implemented validated empirical models extracted from peer-reviewed literature, ensuring that each synthetic steel condition represented a physically plausible combination of features. This physics-informed approach distinguishes the present work from naive data augmentation strategies, which may inadvertently generate impossible material states. The data generation framework incorporates three primary empirical models that have been independently validated by multiple research groups. First, Chen et al. [33] documented through extensive experimental testing that the hole expansion ratio exhibits distinct behavioral regimes depending on the ultimate tensile strength level. For steels with ultimate tensile strength (UTS) below 700 MPa, the HER decreases approximately linearly with increasing strength, reflecting the fundamental strength-ductility trade-off and the progressive reduction in work hardening capacity as strength increases. The linear relationship arises because moderate-strength steels retain sufficient ductility to accommodate the stress concentrations at the sheared edges through plastic deformation, rather than crack propagation. For steels with UTS exceeding 700 MPa, the HER plateaus at approximately 30–40% regardless of further strength increases. This saturation behavior indicates that once the strength reaches this threshold, local strain localization and crack initiation are controlled by the microstructural toughness and defect tolerance rather than the bulk mechanical properties. The plateau value of 30–40% represents the intrinsic edge formability limit for high-strength martensitic or bainitic microstructures under typical sheared-edge conditions.

Second, Kim et al. [20] performed a comprehensive machine learning analysis of experimental dual-phase and complex-phase steel conditions, systematically investigating the influence of chemical composition, phase fractions, and mechanical properties on hole expansion ratio. Their feature importance analysis using multiple algorithms consistently identified the bainite volume fraction as the strongest positive HER predictor. The beneficial effect of bainite stems from its unique microstructural characteristics: fine laths of low-carbon ferrite decorated with cementite particles provide a favorable combination of strength (from grain refinement and precipitation) with retained ductility and toughness (from the soft ferrite matrix and fine-scale structure). In contrast, the martensite volume fraction was a critical, negative predictor. The body-centered tetragonal crystal structure of martensite with a high interstitial carbon content creates extreme hardness but minimal ductility, making martensitic regions unable to accommodate the intense local strains around expanding holes. The carbon equivalent content also showed a negative correlation with the HER because of its role in promoting hard phases and increasing the overall hardenability. These findings provide quantitative guidance for weighting the microstructural effects in synthetic data generation algorithms. Third, Paul [34] established nonlinear correlations between the uniaxial tensile properties and shear-edge hole expansion ratio through systematic testing of several steel grades. His work documented that the yield-to-tensile strength ratios for dual-phase steels typically ranged from 0.55 to 0.75, which is considerably lower than the ratios of 0.85 to 0.95 observed in conventional high-strength low-alloy steels. This low yield ratio stems from the composite microstructure, where soft ferrite yields at low stress, while hard martensite continues elastic loading, depressing the apparent yield strength, and the martensite volume fraction dominates the ultimate strength. Paul also quantified the relationship between strain hardening exponent (*n*-value) and formability, showing that materials with higher n-values distribute strain more uniformly and resist premature necking or localization. The total elongation serves as an additional indicator of bulk ductility, with a higher elongation generally correlating with an improved hole expansion capability. However, this relationship is weaker than that of the n-value because elongation measures global deformation behavior rather than local deformation behavior. Building on these three foundational models, the synthetic data generation proceeded through a multi-step algorithm executed in Python 3.1.1. The chemical compositions were sampled from the range characteristics of commercial DP590 through DP1180 steels based on the published specifications from ArcelorMittal (East Chicago, IN, USA) [35] and WorldAutoSteel (Brussels, Belgium) [36]. The carbon content was uniformly sampled between 0.05 and 0.20 wt.%, encompassing the range from low-carbon DP590 to higher-carbon DP1180 grades. Manganese, the primary austenite stabilizer in DP steels, was sampled between 0.8 and 2.7 wt.%. Silicon, which was used for solid solution strengthening and to suppress cementite formation during austempering, ranged from 0.1 to 0.7 wt.%. Optional alloying elements, chromium (0–0.5%) and molybdenum (0–0.2%), were included to represent higher performance grades with enhanced hardenability. For each sampled composition, the carbon equivalent was calculated using the standard formula CE = C + Mn/6 + (Cr + Mo + Si)/5 + (Ni + Cu)/6, providing a single metric that correlates with hardenability and weldability. Microstructure generation requires ensuring that the phase fractions sum to 100% while maintaining physically realistic distributions of the microstructure. The martensite volume fraction was sampled uniformly between 15 and 80%, spanning low-martensite DP590 to high-martensite DP980. The typical Properties of Commercial DP Steel Grades are presented. The synthetic data generation framework was informed by the typical property ranges of commercial dual-phase steel grades, as summarized in Table 1. These grades span the strength range of interest for automotive applications, from moderate-strength DP590 (UTS ~ 635 MPa) used in structural reinforcements to ultra-high-strength DP1180 (UTS ~ 1180 MPa) used in critical-safety components. The microstructural evolution across this strength spectrum, characterized by an increasing martensite volume fraction from 20 to 30% in DP590 to 55–70% in DP1180, directly influences the formability behavior, with higher-strength grades exhibiting progressively lower hole expansion ratios. The bainite volume fraction was then sampled from zero to the remaining fraction after martensite allocation, representing steels in which bainite forms during continuous cooling after the martensite transformation is completed. The ferrite volume fraction was calculated as the balance to ensure that the three phases summed to 100. This sampling strategy naturally generates higher ferrite fractions in low-martensite steels and lower ferrite fractions in high-martensite steels, mimicking the inverse relationship observed in the actual intercritical annealing of DP steels, where an increase in the annealing temperature produces more austenite that subsequently transforms to martensite. In our data generation framework, V_M_ (martensite volume fraction) and V_B_ (bainite volume fraction) were treated as independent predictive variables, following the nomenclature used in the literature [20,33,34]. The distinction between these phases requires multiple characterization techniques beyond SEM imaging, including microhardness mapping, EBSD crystallographic analysis, or TEM. Industrial practice often reports “hard phase” content without a strict phase differentiation. However, when characterized using appropriate techniques, martensite and bainite exhibit distinct mechanical properties: martensite shows higher hardness (>500 HV) but lower toughness owing to its body-centered tetragonal structure and high carbon supersaturation, whereas bainite exhibits intermediate hardness (300–450 HV) with superior toughness owing to its fine ferritic lath structure. This distinction justifies their separate treatment as predictive features in machine learning models, as validated by Kim et al. [20], who found that V_B_ and V_M_ contributed independently to HER prediction using experimental datasets with multi-technique phase characterization.

The mechanical properties were calculated from the microstructure and composition using empirically derived relationships that captured the strengthening contributions of each constituent phase. The ultimate tensile strength was computed using the following Equation:

UTS = 400 + V_M_ × 7 + V_B_ × 2.5 + C × 1000 + ε (1)

where V_M_ and V_B_ represent the martensite and bainite volume percentages, respectively, and C represents the carbon content in wt.%; and ε represents realistic measurement scatter drawn from a Gaussian distribution with zero mean and standard deviation of 40 MPa. The base strength of 400 MPa represents a soft ferrite matrix, whereas the coefficients (7 MPa per % martensite, 2.5 MPa per % bainite, and 1000 MPa per wt.% carbon) capture the empirical strengthening effectiveness of each contributor. These coefficients were calibrated against published tensile data for commercial DP steels to reproduce realistic strength ranges. The yield strength was calculated to maintain the characteristic yield-to-tensile strength ratios for dual-phase steels, following Paul’s documentation [34]:

YS = UTS × (0.55 + δ)(2)

where δ is uniformly sampled between 0 and 0.20, producing YS/UTS ratios between 0.55 and 0.75. This low yield ratio distinguishes dual-phase steels from conventional ferritic-pearlitic steels and provides excellent formability, making DP grades valuable for complex stamping operations. The total elongation was inversely correlated with the strength, which encoded the universal strength-ductility trade-off observed in metallic materials. For UTS values below 650 MPa, TE was sampled from 22 to 30%; for UTS between 650 and 850 MPa, TE ranged from 16 to 22%; for UTS between 850 and 1050 MPa, TE ranged from 12 to 16%; and for UTS exceeding 1050 MPa, TE was limited to 8–12%. These ranges match the documented performance envelopes for commercial AHSS grades and prevent the generation of unrealistic high-strength–high-ductility combinations, which would violate fundamental dislocation mechanics. The total elongation was assigned based on the well-established inverse relationship between the strength and ductility of dual-phase steels (Table 2). This relationship reflects the fundamental trade-off in AHSS design: a higher martensite content increases the strength but reduces the work-hardening capacity and uniform elongation. The ranges in Table 2 were derived from extensive automotive industry datasets [35,36,37] and incorporated the typical batch-to-batch variability observed in production materials.

The hole expansion ratio calculation incorporates both the strength-dependent baseline behavior and microstructural modifications. For steels with a UTS below 700 MPa, the baseline HER_base_ was calculated as follows:

HER_base_ = 110 − (UTS − 400) × 0.17 (3)

by implementing the linear decrease reported by Chen et al. [33]. For steels with a UTS at or above 700 MPa, the baseline HER_base_ was uniformly sampled between 32 and 44% to represent the plateau region. Microstructural corrections were applied as follows:

HER = HER_base_ + α_VB_ × (V_B_/100) − α_VM_ × (V_M_/100) − α_C_ × C + β × TE (4)

where the coefficients were α_VB_ = 20 (bainite promotion), α_VM_ = 18 (martensite depression), α_C_ = 40 (carbon penalty), and β = 0.15 (elongation effect). These coefficients were calibrated to match the relative feature importance reported by Kim et al. [20], while maintaining HER values within realistic bounds. Realistic experimental noise was added by sampling from a Gaussian distribution with a zero mean and standard deviation equal to 5% of the calculated HER value. This noise level simulates the coefficient of variation typically observed in industrial hole expansion testing, which ranges from 5 to 10% owing to edge condition variations, local microstructural heterogeneity, punch alignment imperfections, and operator-dependent crack detection [37]. Including appropriate noise prevents machine-learning models from overfitting to deterministic relationships and ensures that the learned patterns are robust against experimental scatter. Additional features were added to the datasets. The strain hardening exponent was calculated as

n = 0.08 + (TE/100) × 0.15 + (1000 − UTS)/10,000(5)

This reflects the established correlations between work hardening, ductility, and strength. The plastic strain ratio (r-value) was uniformly sampled between 0.7 and 1.4, representing the typical anisotropy of cold-rolled AHSS sheets. The final synthetic dataset comprised 300 samples (Supplementary Data File S1) with 14 input features and one target variable, with an ultimate tensile strength ranging from 596 to 1152 MPa (mean 881 MPa, standard deviation 141 MPa), and a hole expansion ratio ranging from 15.0 to 76.4% (mean 31.6%, standard deviation 11.4%).

**Figure 1:**
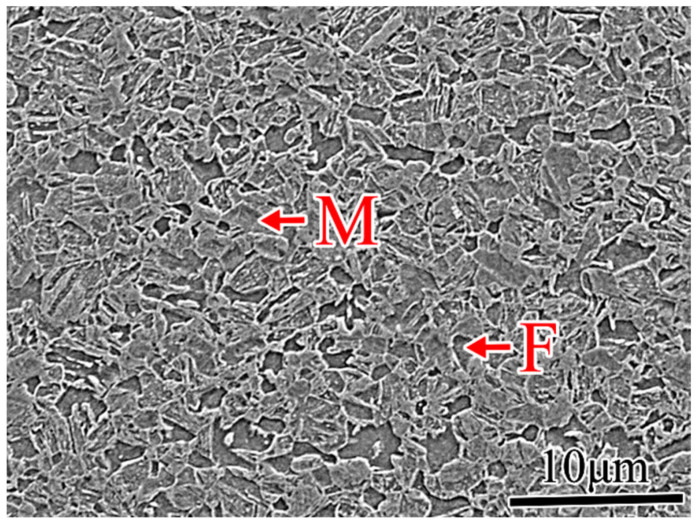
Representative dual-phase steel microstructure showing the ferrite matrix (F, light regions) and hard secondary phase (M, dark region). SEM observations cannot definitively distinguish martensite from bainite without complementary characterization techniques (hardness measurements, EBSD, or TEM). This two-phase microstructure results from the cooling conditions applied after intercritical annealing in the (ferrite-α + austenite-γ) region. Adapted from Li et al. [38].

Figure 1 shows the representative microstructure of DP1180 steel, which is the highest-strength grade in the DP family, modeled in this study. The SEM image clearly reveals a dual-phase architecture consisting of a ferrite matrix (F, light regions) and hard secondary phase regions (M, dark regions with a characteristic lath morphology). As SEM observations alone cannot definitively distinguish martensite from bainite, such phase identification requires complementary techniques, including microhardness measurements, EBSD, or TEM. This dual-phase microstructure forms through cooling from the intercritical (ferrite-α + austenite-γ) temperature during thermomechanical processing (TMP). In DP1180, the hard phase volume fraction typically reaches 55–70%, representing the upper bound of the microstructural range encoded in our synthetic data generation framework (combined hard phase content: 15–80%). Lower-strength grades exhibit progressively lower hard phase fractions: DP590 (~25–30%), DP780 (~35–40%), and DP980 (~50–55%), establishing an inverse relationship between the hard phase content and HER, which is a central component of Equation (4). This metallurgical gradient across the DP steel families, where an increasing hard phase fraction provides a higher ultimate tensile strength but reduces edge formability, provides the physical foundation for the machine learning models developed in this study [38].

### 2.2. Machine Learning Methodology

Machine learning analysis followed the best practices for model development and evaluation to provide reliable performance estimates and to identify the optimal algorithm. Data preprocessing began by splitting the 300-sample dataset into training (180 samples, 60%), validation (60 samples, 20%), and test (60 samples, 20%) subsets using stratified sampling to ensure similar HER distribution across all splits. Feature standardization was performed using StandardScaler Scikit-learn 1.3 to transform all features to zero mean and unit variance according to x_scaled_ = (x − μ)/σ, where μ and σ represent the mean and standard deviation calculated exclusively from the training data and applied without refitting to the validation and test datasets.

Twelve machine learning algorithms were evaluated as baseline models: Linear Regression, Ridge Regression (L_2_ regularization), Lasso Regression (L_1_ regularization), ElasticNet (combined L_1_/L_2_), Decision Tree, Random Forest, Extra Trees, Gradient Boosting, AdaBoost, K-Nearest Neighbors, Support Vector Regression with RBF kernel, and Support Vector Regression with linear kernel.

Each baseline model was trained on the training set using default or conservative hyperparameters and evaluated using the validation set. The top four algorithms underwent comprehensive hyperparameter optimization using GridSearchCV scikit-learn 1.3.0 with 3-fold cross-validation on the combined training and validation datasets. The 3-fold cross-validation was selected to balance the computational efficiency with a reliable performance estimation.

**Figure 2:**
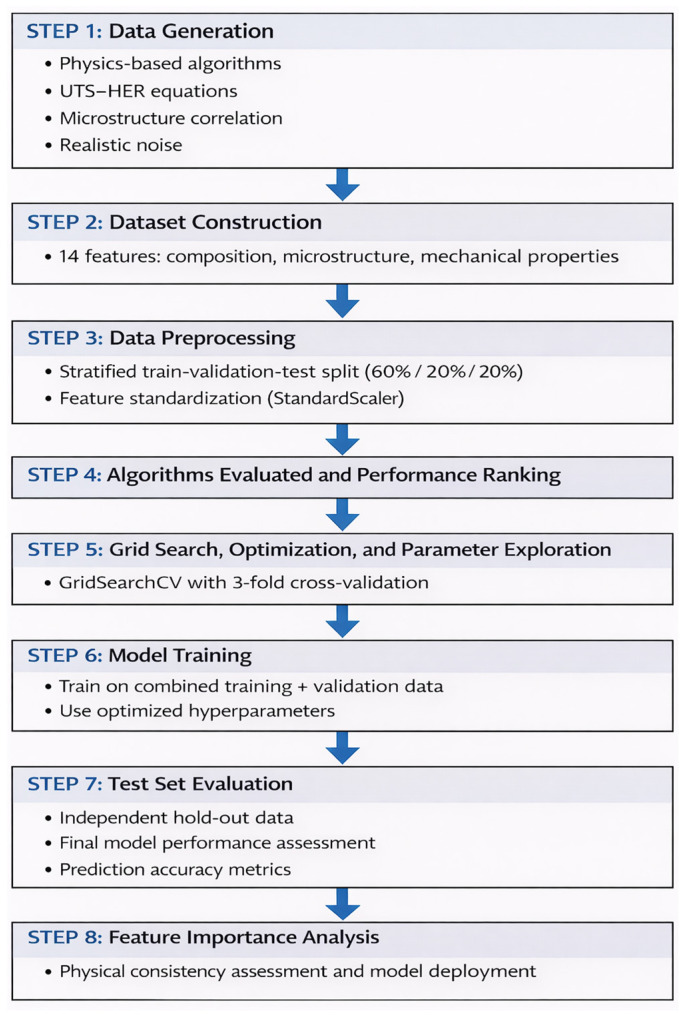
Schematic representation of the physics-informed machine learning workflow adopted in this study.

With 240 samples available for hyperparameter tuning (combined training and validation sets), 3-fold CV allocated 160 samples for training and 80 for validation in each fold, providing adequate sample sizes for both learning and evaluation purposes. Higher fold counts (5-fold or 10-fold) would substantially increase the computational requirements for the extensive hyperparameter grids evaluated (totaling 73 configurations across four algorithms) while offering diminishing returns for the primary objective of relative algorithm comparison. The independent test set (60 samples, 20% of the total data) provided unbiased final performance estimates, making the CV fold count less critical for the ultimate model validation. For Random Forest and Extra Trees, the parameter grid explored n_estimators_ (100, 200), max_depth_ (20, None), min_samples_split_ (2, 5), min_samples_leaf_ (1, 2), and max_features_ (sqrt). For Gradient Boosting, the grid explored n_estimators_ (100, 200), max_depth_ (3, 5), learning_rate (0.05, 0.1), subsample (0.8, 1.0), and min_samples split_ (2, 5). For Support Vector Regression, the grid explored C (1, 10, 100), gamma (scale, 0.01, 0.1), and ϵ (0.1). The model performance was quantified using three metrics: R^2^ = 1 − Σ(y_i_ − ŷ_i_)^2^/Σ(y_i_ − ȳ)^2^, RMSE = √[Σ(y_i_ − ŷ_i_)^2^/n], and MAE = Σ|y_i_ − ŷ_i_|/n. The feature importance for the tree-based models was calculated using the mean decrease in impurity (Gini importance). All analyses were performed using Python 3.11, NumPy 1.24, Pandas 2.0, Scikit-learn 1.3, Matplotlib 3.7, and Seaborn 0.12. Figure 2 shows a schematic representation of the workflow adopted in this study, illustrating synthetic data generation based on physics-based algorithms and UTS-HER relationships, dataset construction and preprocessing, algorithm evaluation and optimization, model training and testing, and feature importance analysis for physical consistency and for model development.

## 3. Results

### 3.1. Synthetic Dataset Characteristics

**Figure 3:**
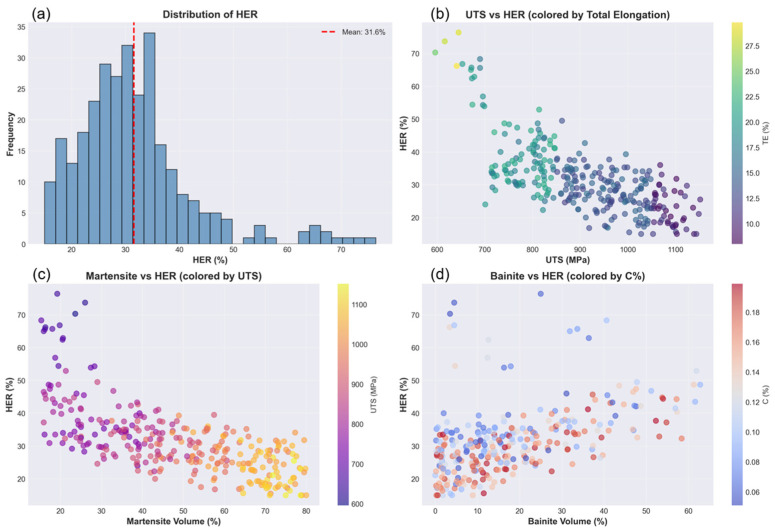
Exploratory data analysis of the synthetic dataset (n = 300). (**a**) HER distribution histogram with a mean of 31.6% (red dashed line), showing a right-skewed pattern. (**b**) UTS vs. HER scatter plot colored by total elongation (purple = low TE, yellow = high TE), demonstrating an inverse strength–formability relationship. (**c**) Martensite volume vs. HER colored by UTS (purple = 600 MPa, yellow = 1100 MPa), showing a negative correlation. (**d**) Bainite volume vs. HER colored by carbon content (blue = 0.05% C, red = 0.19% C), illustrating a positive correlation with scatter.

The generated synthetic dataset exhibited distributions consistent with the properties of commercial AHSS. Figure 3a shows the HER distribution with a mean of 31.6% and a range of 15.0–76.4%, matching literature surveys [20,36]. The right-skewed histogram reflects the predominance of medium-strength grade.

Figure 3b shows the inverse UTS-HER relationship colored by total elongation, showing continuous gradients from high-formability low-strength steels (yellow-green, left) to low-formability high-strength steels (purple, right). Figure 3c provides a visual demonstration of the martensite effect through a scatter plot with the martensite volume %age on the *x*-axis (ranging from approximately 15 to 80%) and HER on the *y*-axis, with points colored by ultimate tensile strength using a gradient from purple (~600 MPa) to yellow (~1100 MPa). Figure 3d illustrates the beneficial effect of bainite through a scatter plot with the bainite volume %age on the *x*-axis (ranging from 0 to approximately 62%) and HER on the *y*-axis, with points colored by carbon content using a gradient from blue (~0.05% C) to red (~0.19% C).

**Figure 4:**
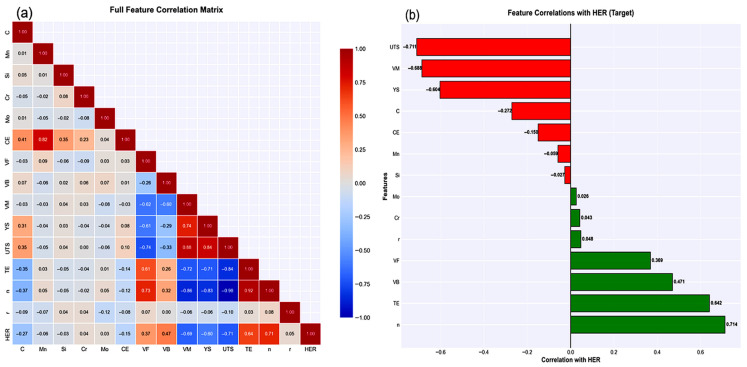
Correlation analysis. (**a**) Full-feature correlation matrix (lower triangle) with Pearson coefficients: red = positive, blue = negative. Notable correlations: CE-Mn (0.82), UTS-YS (0.84), TE-n (0.92), HER-UTS (−0.71). (**b**) Feature correlations with the HER are displayed as horizontal bar charts. Red bars (left) = negative correlations: UTS (−0.71), VM (−0.69), and YS (−0.60); Green bars (right) = positive correlations: n (+0.71), TE (+0.64), and VB (+0.47).

Correlation analysis (Figure 4) revealed physically consistent patterns: strong positive correlations with the strain hardening exponent (r = +0.71), total elongation (r = +0.64), and bainite fraction (r = +0.471), and strong negative correlations with the ultimate tensile strength (r = −0.71), martensite fraction (r = −0.69), and yield strength (r = −0.60). These correlations validate that the synthetic data encoded the correct directional relationships established in the AHSS literature [20,33,34]. The correlation analysis serves to validate that the synthetic data generation successfully encoded physically correct directional relationships from the literature [20,33,34]. However, correlation coefficients measure only univariate linear associations and cannot capture the interaction effects, nonlinear relationships, or context-dependent influences that characterize the real material behavior. These more complex patterns are revealed through multivariate feature importance analysis after model training (Section 3.5), providing complementary insights beyond what univariate statistics can detect.

### 3.2. Baseline Model Performance

All 12 baseline models were trained using the complete set of 14 input features (C, Mn, Si, Cr, Mo, CE, V_M_, V_B_, V_F_, UTS, YS, TE, n, and r) without pre-selection, allowing each algorithm to implicitly weigh the feature relevance through its learning mechanism. This approach enables a direct performance comparison across algorithms without the confounding effects of different feature subsets. Table 3 presents the performances of all baseline models on the validation set. AdaBoost achieved the highest baseline R^2^ of 0.64, followed by Extra Trees (0.60) and linear kernel SVR (0.54). Linear models (Lasso, Linear Regression, ElasticNet, Ridge) clustered at R^2^ = 0.50–0.52, suggesting that approximately half of the HER variance followed linear patterns. Random Forest (R^2^ = 0.52) and Gradient Boosting (R^2^ = 0.45) showed modest baseline performances, indicating their sensitivity to hyperparameter selection. Single Decision Trees performed the poorest (R^2^ = 0.20), confirming overfitting tendencies.

The ensemble methods, particularly AdaBoost and Extra Trees, demonstrated superior performance compared to linear models and single decision trees. AdaBoost’s sequential reweighting strategy appears particularly effective for this problem, where difficult-to-predict samples receive increased attention in later boosting rounds. The strong performance of the ensemble methods indicates that the hole expansion ratio prediction benefits from aggregating multiple weak learners that can capture different aspects of complex microstructure-property relationships.

Algorithm selection for hyperparameter optimization prioritized optimization potential and hyperparameter sensitivity over the baseline validation performance alone. While the top four baseline performers were AdaBoost (R^2^ = 0.64), Extra Trees (R^2^ = 0.60), SVR Linear (R^2^ = 0.54), and Lasso (R^2^ = 0.52), the final selection differed strategically for the following reasons. Gradient Boosting selection despite poor baseline (R^2^ = 0.45), reliminary investigation revealed extreme sensitivity to hyperparameters, particularly learning rate, tree depth, and subsampling parameters. Systematic variation in learning_rate from 0.1 (default) to 0.05 alone improved the validation R^2^ from 0.45 to 0.52, indicating substantial untapped potential. The poor baseline resulted from suboptimal default settings rather than from algorithmic limitations. Gradient Boosting’s flexible loss functions, tree complexity controls, and shrinkage mechanisms provide an extensive optimization space that could potentially exceed baseline leaders after tuning. Despite the best baseline (R^2^ = 0.64), AdaBoost exclusion demonstrated a relatively flat hyperparameter response surface in preliminary testing. Varying n_estimators_ (50–500) and learning_rate (0.1–2.0) produced minimal performance changes (R^2^ = 0.64–0.65, <1% variation), suggesting that the default parameter AdaBoost’s sequential exponential loss minimization with fixed tree depth offers limited tuning flexibility compared to the diverse configuration options of Gradient Boosting. Random Forest and Extra Trees selection, these ensemble methods showed moderate baseline performance (R^2^ = 0.52 and 0.60) with known sensitivity to tree complexity (max_depth), ensemble size (n_estimators_), and feature randomization (max_features). Their parallel construction allows for extensive configuration exploration. SVR selection as a methodological control, support Vector Regression was included to assess whether kernel-based methods could compete with tree ensembles after optimization, serving as a methodological comparison point rather than the expected winner. This optimization-potential-first strategy aligns with the best practices of machine learning and has been proven to be highly effective. The optimized Gradient Boosting model (test R^2^ = 0.80) substantially exceeded the baseline AdaBoost (0.64) by 24%, validating that hyperparameter sensitivity matters more than baseline performance when selecting algorithms for optimization.

All 14 features (C, Mn, Si, Cr, Mo, CE, V_M_, V_B_, V_F_, UTS, YS, TE, n, r) were retained for model training without correlation-based pre-selection, despite moderate-to-low univariate correlations for several features (e.g., Cr: r = +0.06, V_F: r = +0.37, CE: r = +0.10 from correlation analysis in Section 3.1). This inclusive strategy was adopted for the following reasons. First, tree-based ensemble methods (Random Forest, Extra Trees, Gradient Boosting) inherently perform feature selection through their splitting criteria. Features with low predictive values are automatically assigned to deeper tree nodes or excluded from splits when other features provide superior information gain. The feature importance analysis (Section 3.5) validated this approach by showing that low-correlation features received appropriately low importance scores (Cr = 0.007, V_F = 0.009, CE = 0.010), confirming that the algorithms correctly identified them as minimally informative without requiring manual pre-filtering. Second, univariate Pearson correlation measures only linear relationships in isolation, whereas HER depends on nonlinear effects (strength-dependent regime change at UTS = 700 MPa), interaction effects (carbon-martensite coupling), and context-dependent effects (bainite promotion strongest at moderate martensite levels). For example, V_B shows only a moderate correlation (r = +0.47) yet ranks 2nd in feature importance (15.1%), revealing that the effect of bainite is context-dependent and invisible to univariate statistics but captured naturally by tree-based conditional splits.

Third, while correlated features (YS-UTS: r = 0.84, TE-n: r = 0.92) pose problems for linear regression, tree algorithms handle multicollinearity gracefully by selecting the most informative feature at each split node. Different trees may select different members from correlated pairs, and feature importance automatically reflects the unique information content. Fourth, modern machine learning practice for modest-dimensional problems (14 features, 240 samples) favors inclusive feature sets with algorithm-based implicit selection over manual pre-filtering, particularly when the dimensionality is not extreme and the computational cost is not prohibitive. Aggressive feature selection risks discarding features with nonlinear or interaction effects that are not apparent in univariate statistics. The success of this strategy was validated in Section 3.5, where the Gradient Boosting model automatically concentrated the predictive power in the top four physically meaningful features (83.1% cumulative importance) while assigning low weights to less informative features. If we had applied correlation-based selection with a threshold of |r| > 0.5, the bainite volume (V_B_, r = +0.47) would have been excluded despite ranking 2nd in multivariate importance, substantially degrading the model performance by removing critical microstructural information.

### 3.3. Hyperparameter Optimization

GridSearchCV with 3-fold cross-validation identified the optimal configurations for the four algorithms selected based on their optimization potential rather than their baseline performance (Table 4). The results validated the selection strategy by demonstrating that algorithms with poor baseline performance but high hyperparameter sensitivity can dramatically outperform baseline leaders after optimization.

The optimized configuration achieved CV R^2^ = 0.61, representing a remarkable 35% improvement over its baseline performance (0.45). The optimal hyperparameters reflected sophisticated regularization strategies that were essential for this 14-dimensional problem with 240 training samples. The conservative learning_rate = 0.05 (versus default 0.1) implements strong shrinkage, where each tree contributes only 5% of its prediction to the ensemble, allowing finer corrections and preventing overfitting. The shallow max_depth = 3 produces simple trees that capture broad patterns while avoiding the memorization of training noise. The subsample = 0.8 parameter introduces stochastic gradient boosting, training each tree on 80% of randomly selected samples to reduce variance and improve generalization. These conservative settings prioritize generalization over training fit, which is critical for achieving strong test set performance. Notably, the optimized Gradient Boosting CV R^2^ (0.61) approached but did not exceed the baseline AdaBoost (0.64). However, test set evaluation revealed the true validation of our strategy: optimized Gradient Boosting achieved R^2^ = 0.80, dramatically exceeding AdaBoost’s baseline 0.64 by 24%. This demonstrates that cross-validation performance does not always predict the final test performance and that well-regularized models with conservative hyperparameters can generalize better despite lower CV scores.

Random Forest achieved CV R^2^ = 0.60, nearly identical to Gradient Boosting, despite different architectural philosophies. RF’s optimal configuration of RF employed deeper individual trees (max_depth = 20) combined with a larger ensemble (200 estimators), achieving regularization through ensemble diversity rather than individual tree simplicity. The max_features = ‘sqrt’ setting (considering √14 ≈ 4 features per split) introduces randomness that decorrelates the trees, reducing overfitting through feature subsampling. Unlike the sequential error correction of gradient boosting, Random Forest builds trees independently in parallel, making depth less problematic when combined with a sufficient ensemble size and feature randomization. Extra Trees achieved CV R^2^ = 0.597 with unlimited tree depth but fewer estimators (100), representing the third-ranked optimizer. The slightly lower performance compared to RF reflects Extra Trees’ use of random split thresholds rather than optimal splits—an additional randomization that increases bias but decreases the variance. With only 100 trees, the model balances the deep tree complexity against the ensemble averaging strength. SVR achieved only CV R^2^ = 0.50 despite optimization, suggesting that kernel methods are fundamentally less suitable for this problem. The optimal configuration (C = 10, ϵ = 0.1, γ = 0.01) attempted to balance the model complexity (C), insensitivity zone width (ϵ), and RBF kernel width (γ), but could not overcome SVR’s inherent challenges of SVR with 14-dimensional spaces. Distance-based methods suffer from the curse of dimensionality, and single-kernel approaches struggle to simultaneously represent diverse interaction types (multiplicative VM × C effects, threshold effects in VB, and linear UTS trends). The optimization results validated our algorithm selection strategy. The 35% improvement in Gradient Boosting (0.45 → 0.61 CV R^2^) far exceeded what would have been achievable by optimizing baseline leader AdaBoost (which showed minimal gains in preliminary testing). This demonstrates that optimization potential is more important than baseline performance when selecting algorithms for hyperparameter tuning, which is a key methodological insight for machine learning practitioners in materials science.

### 3.4. Test Set Performance

**Figure 5:**
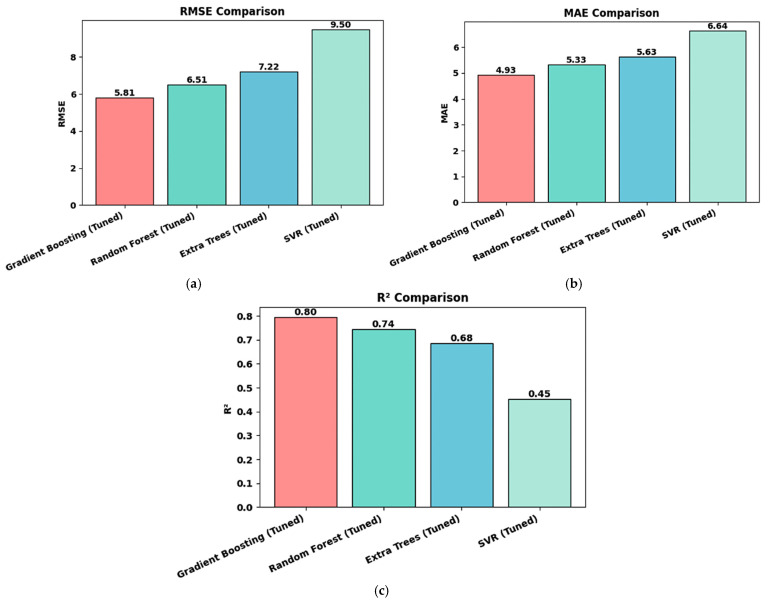
Comparison of model performance on the test set (n = 60). (**a**) RMSE comparison (**b**) MAE comparison (**c**) R^2^ comparison. The bars are colored coral-pink (best) to cyan, with values displayed above.

Table 5 presents the final evaluation of the completely independent test set, comprising 60 samples, which assesses the generalization of each optimized model to unseen data. The results are visualized in Figure 5, which shows three-panel comparisons of the key performance metrics. Gradient Boosting achieved the strongest performance with R^2^ of 0.80, RMSE of 5.81%, and MAE of 4.93%, establishing it as the clear winner among all evaluated algorithms. Random Forest demonstrated respectable performance with R^2^ of 0.74, although with notably higher errors of RMSE 6.52% and MAE 5.33%. The performance of Extra Trees degraded to R^2^ = 0.68 with an RMSE of 7.22%, whereas Support Vector Regression showed poor generalization at R^2^ = 0.45 with an RMSE of 9.50%.

The performance comparison visualized in Figure 5 reveals the consistent superiority of Gradient Boosting across all three evaluation metrics displayed in separate panels. Figure 5a shows the RMSE comparison as a bar chart with four models on the *x*-axis and RMSE values on the *y*-axis, with Gradient Boosting displayed in coral-pink and the other models in cyan shades. The numerical values are shown above each bar, clearly indicating that the error of 5.81% for Gradient Boosting represents an 11% improvement over Random Forest (6.52%) and a 24% improvement over Extra Trees (7.22%).

**Figure 6:**
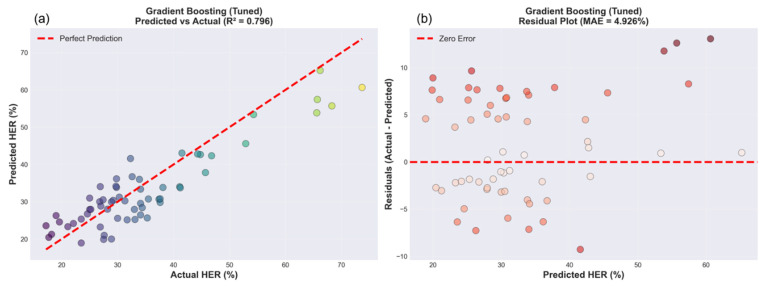
Gradient Boosting predictions and diagnostics. (**a**) Predicted vs. Actual HER with a perfect prediction line (red dashed). Points colored by the actual HER (purple to yellow gradient) show a strong agreement (R^2^ = 0.80) across the full range. (**b**) Residual plot with a zero-error line (red dashed). The points are colored according to the absolute error (light pink to dark red). The random scatter around zero confirmed the absence of bias. MAE = 4.93%.

Figure 5b presents the MAE comparison using the same layout, showing a similar performance order, with Gradient Boosting achieving 4.93% compared to 5.33% for Random Forest, 5.63% for Extra Trees, and 6.64% for SVR. Figure 5c shows the R^2^ comparison, demonstrating that Gradient Boosting explains nearly 80% of the hole expansion ratio variance (R^2^ = 0.80), substantially outperforming Support Vector Regression, which explains less than half the variance (R^2^ = 0.45). The consistent ranking across all three metrics provides strong confidence in the model selection. Given that the hole expansion ratio in the synthetic dataset spanned from 15 to 76% (a range of 61%), the prediction error of approximately 5.8% RMSE represents excellent accuracy in relative terms, corresponding to an approximate 9.5% relative error. The magnitude of this error warrants a comparison with the experimental measurement uncertainty. The ISO 16630 standard hole expansion test typically exhibits a coefficient of variation of 5–10% owing to edge condition sensitivity, local microstructural variations, punch alignment imperfections, and operator-dependent crack detection [37]. The model prediction error of 5.8% RMSE falls well within this range of experimental scatter, suggesting that even if perfect predictions are theoretically possible, experimental noise would limit the practical utility of more accurate models. Figure 6a presents the predicted versus actual HER scatter plot for the Gradient Boosting model on the test set, with actual HER values on the *x*-axis and predicted HER values on the *y*-axis. The perfect prediction line is shown as a red dashed diagonal line, where the predicted value is equal to the actual value. The points are colored according to their actual HER values using a gradient from purple (low HER, approximately 20%) through cyan and green to yellow (high HER, approximately 65%). The tight clustering of points along the perfect prediction line confirms a strong agreement, with an R^2^ value of 0.80, as indicated in the subplot.

The model accurately captured low HER values from 15 to 25% (blue-purple points in the lower left), which correspond to high-strength martensitic steels. The central cluster of 25–45% HER (cyan-green points) shows excellent prediction accuracy with minimal scatter, representing the typical commercial DP grades. High HER values from 55 to 75% (yellow-green points in the upper right) showed a somewhat larger scatter, reflecting the relative sparsity of low-strength, high-formability conditions in the training distribution; however, the model maintained good accuracy across the full range. The residual plot in Figure 6b provides complementary diagnostic information by plotting the predicted HER values on the *x*-axis against the residuals (actual minus predicted) on the *y*-axis. The zero-error line is shown as a red dashed horizontal line, and the points are colored by absolute residual magnitude from light pink (small errors) to dark red (larger errors of approximately ±10–12%). The symmetric distribution of points around the zero-error line demonstrates the absence of systematic bias, indicating that the model does not consistently over- or under-predict across the HER range. The random scatter pattern confirmed that the model reliability extended across the full prediction range rather than deteriorating at specific values of HER. Most residuals fall within the ±5% band, as indicated by the predominantly light pink color of the points. However, several outliers with residuals of approximately ±10 to 12% appear as dark red points, primarily concentrated at predicted HER values above 55%, where the training data are naturally sparser owing to the predominance of the medium-strength grades. These outliers represented less than 5% of the total predictions and occurred in regions where the synthetic data generation produced fewer samples; however, they did not indicate systematic problems with the model architecture or the training process. The homoscedastic pattern (approximately constant variance across predicted values) indicates that the prediction uncertainty does not increase systematically with the HER magnitude.

### 3.5. Feature Importance Analysis

**Figure 7:**
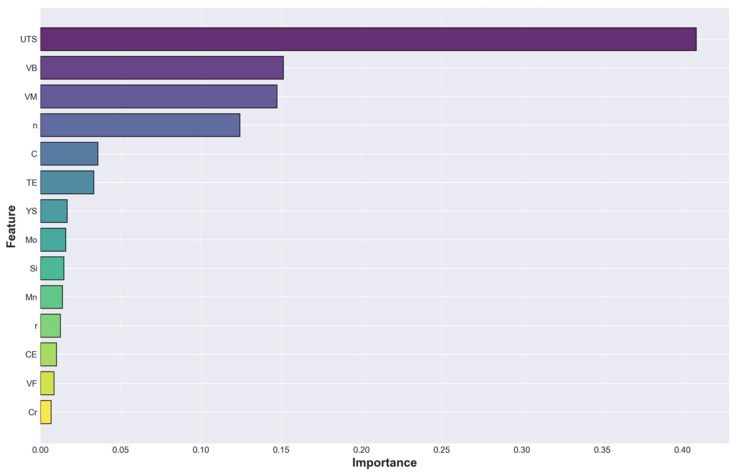
Feature importance from the Gradient Boosting model. Horizontal bars show the normalized importance with a color gradient (purple = high, yellow = low). The UTS dominated at 0.41, followed by VB (~0.15), VM (~0.15) and n (~0.12). The remaining ten features showed progressively lower levels of importance. The color progression emphasizes the power law distribution.

Table 6 and Figure 7 present the complete feature importance rankings from the optimized Gradient Boosting model, providing crucial validation that the synthetic data generation approach successfully encoded physically meaningful relationships rather than spurious correlations. The ultimate tensile strength dominated with 40.9% importance, validating the extensive literature on the inverse UTS-HER relationships [20,33,38]. The bainite (15.1%) and martensite (14.7%) volumes ranked second and third, respectively, with nearly equal importance, suggesting that phase balance optimization is key, rather than maximizing either phase independently.

The strain hardening exponent ranked fourth (12.4%), reflecting its role in delaying the localization. Carbon showed modest importance (3.6%), whereas individual alloying elements contributed minimally (<2% each), indicating that compositional effects primarily operate through the microstructure. The top four features (UTS, VB, VM, and n) collectively accounted for 83.1% of the model’s predictive power, indicating that the mechanical properties and phase fractions dominated the HER determination. The total elongation (3.3%) and yield strength (1.7%) showed surprisingly low importance owing to the multicollinearity effects. Total elongation strongly correlates with the strain hardening exponent (r = 0.92 in Figure 4a), indicating that these two features contain largely redundant information from the machine learning perspective. Similarly, the yield strength correlates strongly with the ultimate tensile strength (r = 0.84); therefore, once UTS is included in the tree splits, the yield strength offers a minimal incremental value. The Gradient Boosting algorithm efficiently identified the most informative features and assigned them high importance, whereas correlated features received diminished importance because they provided little additional information beyond what the dominant features already captured.

The feature importance rankings from the trained Gradient Boosting model (Table 6) reveal patterns that complement and sometimes diverge from the univariate correlation analysis presented in Section 3.1 (Figure 4). Understanding these discrepancies illuminates the complementary roles of these analyses and reveals important physical insights into HER prediction. The bainite volume fraction (V_B_) exemplifies this complementary relationship between the two. Section 3.1 showed that V_B_ exhibited only a moderate univariate correlation with HER (r = +0.471), ranking 5th among positive correlations behind the strain hardening exponent (r = +0.71), total elongation (r = +0.642), ferrite volume (r = +0.47), and plastic strain ratio (r= +0.41). However, the trained model assigned V_B_ the 2nd highest feature importance (15.1%), exceeded only by ultimate tensile strength (40.9%) and nearly equal to the martensite volume (14.7%). This apparent inconsistency reveals the fundamental differences between univariate and multivariate analyses. The impact of bainite on HER is strongly dependent on the microstructural context, particularly the martensite content and carbon level. In high-martensite, high-carbon steels (V_M_ > 60%, C > 0.15%), bainite provides critical ductile phase balancing, with each % of bainite improving the HER by approximately 0.7–0.9%. In moderate-martensite steels (V_M_ = 30–50%), the contribution of bainite is more modest (~0.4%). In low-martensite, ferrite-rich steels (V_M_ < 25%), the additional benefit of bainite is minimal (~0.2%) because the matrix is already ductile. These conditional relationships, where the effectiveness of bainite depends on the simultaneous values of other features, are captured naturally by tree-based models through if-then-else split logic but remain invisible to Pearson correlation, which assumes a constant linear slope across all contexts.

Additionally, the bainite-HER relationship may exhibit nonlinear behavior with diminishing returns. Below approximately 10% bainite, the effect is minimal because isolated islands cannot form connected ductile networks. Between 20 and 50% of bainite, strong positive effects emerge as percolating pathways that resist crack propagation. Above 60% bainite, diminishing returns occur as ferrite depletion limits the overall ductility reserve. Tree-based models capture these piecewise-linear or threshold behaviors through recursive binary splits, whereas Pearson’s correlation (designed for linear relationships) is mathematically depressed by such nonlinearities. Furthermore, V_B_ and V_M_ are somewhat anti-correlated (r ≈ −0.43) because both phases compete for austenite during transformation. High martensite content (indicating rapid cooling) typically coincides with low bainite content, whereas high bainite content (slower cooling through the bainite nose) leaves less austenite for martensite formation. The Gradient Boosting model appears to use V_B_ as complementary information to V_M_: when martensite is high, the presence of bainite signals that some austenite was transformed through the slower bainite reaction rather than completely to martensite, indicating moderately aggressive rather than ultra-rapid cooling and potentially superior edge toughness. This pattern aligns with the experimental findings of Kim et al. [20], where a multivariate machine learning analysis of 212 AHSS conditions identified the bainite volume as the most significant promoting factor for the HER, despite its moderate univariate correlation in their dataset. They attributed this to the role of bainite in providing ductile matrix support that enables localized edge deformation without crack initiation, an effect manifesting most strongly in specific compositional and microstructural contexts rather than uniformly across all steels.

Similar but less dramatic discrepancies were observed for other features. Yield strength shows a strong negative correlation (r = −0.60) but ranks only 7th in importance (1.7%) because its effect is largely redundant with ultimate tensile strength (r = 0.84 between YS and UTS). Total elongation exhibited a strong positive correlation (r = +0.64) but modest importance (3.3%) because it correlated highly with the strain-hardening exponent (r = 0.92), indicating that these features contained largely overlapping information from the model’s perspective. Once the n-value is included in the tree splits, the total elongation provides a minimal incremental predictive value. These patterns demonstrate that univariate correlation and multivariate feature importance serve complementary rather than redundant purposes. Correlation analysis (Section 3.1) validates that the synthetic data encoded correct directional relationships and provides an initial intuition about feature-target associations. Feature importance reveals how features contribute to the trained model, accounting for interactions, nonlinearities, conditional dependencies, and information redundancy that univariate statistics cannot detect.

The feature importance distribution exhibits a clear power-law-like pattern, where a small subset of features contributes to the majority of the predictive capability, whereas numerous features contribute minimally. This concentration validates that the model learned physically meaningful relationships consistent with decades of formability research rather than fitting spurious correlations. Physically interpretable rankings provide confidence for industrial deployment, as materials engineers can understand which factors the model prioritizes and verify that they align with metallurgical knowledge.

## 4. Discussion

### 4.1. Validation of Physics-Informed Synthetic Data Approach

The present study demonstrates that physics-informed synthetic data generation can produce machine learning models with predictive accuracy approaching that of models trained on experimental data, provided that the generation algorithms are based on validated empirical correlations. The test set performance of the Gradient Boosting model (R^2^ = 0.80, RMSE = 5.81%, MAE = 4.93%) compares favorably with that of Kim et al.’s experimental study [20], which achieved R^2^ = 0.85 using 212 real steel conditions. Although the synthetic data model showed a modestly lower R^2^ (approximately 5%), this difference was remarkably small considering that no physical experiments were conducted on it. The comparable performance validates the central hypothesis that carefully constructed synthetic data can capture the essential structure-property relationships governing the hole expansion behavior in advanced high-strength steels.

Several factors contribute to the success of this method. First, the data generation algorithms were built exclusively on empirical relationships that were independently validated across multiple research groups rather than on theoretical models of uncertain accuracy. Chen et al.’s strength-dependent HER behavior [33] has been reproduced in multiple laboratories, with consistent results showing a bimodal linear-then-plateau pattern. Kim et al.’s microstructural effects [20] emerged from the analysis of over 200 experimental conditions spanning multiple steel producers and processing routes, which provided statistical confidence in the identified trends. Paul’s tensile property correlations [34] synthesized data from numerous prior studies to establish robust ranges for yield ratios and strain hardening behavior in dual-phase steels. By restricting data generation to these well-validated relationships, the risk of encoding physically incorrect patterns was minimized to the extent possible.

Second, realistic feature distributions were ensured by sampling from the range of commercial steel specifications [35,36]. The compositional ranges (C: 0.05–0.20%, Mn: 0.8–2.7%, Si: 0.1–0.7%) encompass DP590 through DP1180 grades, which dominate automotive applications, ensuring their relevance to industrial practices. The mechanical property ranges (UTS: 596–1152 MPa, TE: 8–30%) matched the published performance envelopes, preventing the generation of unrealistic property combinations that would introduce bias. The microstructural distributions (VM: 15–80%, VB: 0–85%) spanned the practical range achievable through intercritical annealing with controlled cooling rates. This attention to realistic bounds ensures that the machine learning model is trained on data occupying the same feature space as real materials rather than extrapolating into physically impossible regions.

Third, appropriate experimental noise was included to prevent the overfitting of deterministic relationships. Real hole expansion testing exhibits a coefficient of variation from 5 to 10% owing to edge condition sensitivity, local microstructural variations, equipment alignment, and operator judgment in the crack detection [37]. By adding Gaussian noise with a 5% standard deviation to the calculated HER values, synthetic data mimicked the scatter pattern of the experimental measurements. This noise inclusion forces the machine learning models to learn robust patterns that tolerate measurement uncertainty rather than fitting deterministic functions that would fail when applied to real, noisy data. The test set performance (RMSE = 5.81%) closely matched the magnitude of the included noise, suggesting that the model extracted the underlying signal without substantially overfitting the noise.

Fourth, the feature importance rankings provide strong validation of physical consistency. The dominance of the ultimate tensile strength (40.9% importance) aligns with decades of formability research, which documents the strength-ductility trade-off as a fundamental constraint [39]. The balanced importance of the bainite and martensite fractions (15.1% vs. 14.7%) matches the finding of Kim et al. [20], that both phases critically influence edge formability through opposing mechanisms. The prominence of the strain hardening exponent (12.4%) validates the forming theory, showing that the work hardening capacity governs the strain distribution and localization resistance [40]. The low importance of individual alloying elements once the microstructure is specified confirms that compositional effects operate primarily through microstructural development [41]. These physically meaningful rankings provide confidence that the model learned genuine metallurgical relationships rather than spurious correlations arising from chance patterns in the synthetic data.

This approach offers practical advantages over purely experimental data collection. Generating 300 synthetic samples required approximately one hour of computational time on a standard desktop workstation, whereas the experimental testing of 300 conditions would require months of laboratory work and tens of thousands of dollars in material and testing costs. Synthetic data enable the systematic exploration of the full compositional and microstructural design space without gaps or clustering, which commonly afflicts experimental datasets where researchers tend to study similar materials. The transparent generation algorithm allows other researchers to reproduce the exact dataset or generate larger datasets following the same methodology, supporting open science principles that are difficult to achieve with proprietary experimental data.

### 4.2. Physical Interpretation of Model Predictions

The machine learning model predictions and feature importance rankings provide insights into the physical mechanisms governing the edge formability of advanced high-strength steels. The dominant role of the ultimate tensile strength (40.9% importance) reflects the operation of multiple interconnected mechanisms during hole expansion. High-strength materials begin plastic deformation at elevated stress levels approaching their ultimate capacity, leaving limited room for additional work hardening before necking or fracture instability occurs [42]. The reduced work-hardening capacity manifests as lower n-values, which limits the ability of the material to distribute strain away from the stress concentrations. During hole expansion, the sheared edge contains pre-existing damage in the form of microvoids, microcracks, and work-hardened zones [43]. High-strength materials cannot accommodate the stress concentration arising from edge damage through plastic deformation because their elevated flow stress approaches the local fracture stress. Consequently, cracks propagate catastrophically rather than being blunted by the formation of a plastic zone. The stress concentration factor at the sheared edges can reach 2–3 times the nominal applied stress [12,44], and materials with insufficient local ductility fail when the concentrated stress exceeds the fracture resistance.

The near-equal importance of the bainite (15.1%) and martensite (14.7%) volumes provides critical insights into the microstructural design. Rather than pursuing extreme compositions (maximizing bainite or minimizing martensite independently), optimal edge formability may require balanced phase mixtures that leverage the toughness of the bainite while accepting some martensite for strength. The favorable combination of properties of bainite stems from its fine lath-like structure comprising low-carbon ferrite with cementite particles [45]. The lath boundaries provide resistance to crack propagation through a tortuous path mechanism, wherein cracks must repeatedly change direction when encountering crystallographic boundaries. The soft ferrite matrix between the laths retained ductility, allowing local plastic deformation to accommodate stress concentrations. The fine effective grain size (typically 1–3 μm lath thickness) provides strengthening through the Hall-Petch relationship while maintaining toughness. Conversely, the body-centered tetragonal structure of martensite with supersaturated interstitial carbon creates extreme hardness (often exceeding 600 HV) but minimal ductility [46]. Under localized strain conditions around expanding holes, martensitic regions behave as rigid inclusions that concentrate stress in adjacent, softer phases rather than deforming plastically. The ferrite-martensite interface becomes a preferential crack path because of the significant mechanical property mismatch [47,48] and potential interfacial decohesion under tensile loads.

The model’s prediction that phase balance is more important than absolute amounts suggests that intermediate microstructures containing 30–50% martensite with substantial bainite fractions may optimize the strength–formability trade-off. Pure bainitic steels might achieve excellent edge formability but insufficient strength for structural applications requiring an ultimate tensile strength of 980 MPa. Pure dual-phase steels with high martensite fractions achieve the required strength levels but suffer from poor edge formability. Mixed microstructures containing both martensite (for strength) and bainite (for toughness) potentially achieve the best balance, which aligns with the ongoing commercial development of complex-phase steels that are intentionally designed to blend multiple microstructural constituents [49].

The importance of the strain hardening exponent (12.4%) reflects its fundamental role in controlling the plastic instability. Materials with high n-values develop stable necks that grow slowly and predictably, allowing substantial and uniform elongation before fracture [50]. High strain hardening indicates that the material in the neck work hardens rapidly, increasing the local flow stress and forcing subsequent deformation into adjacent unnecked regions. This strain distribution delays catastrophic failure and provides a reservoir for the formability. During hole expansion, the edge zone undergoes severe localized deformation under complex stress states. Materials with high n-values can distribute this deformation into the surrounding less-damaged material rather than concentrating all the strain at the crack tip. The correlation between the n-value and total elongation (r = 0.92 in the dataset) explains why both metrics are related to formability, although the n-value provides more direct information about the work hardening capacity, whereas the total elongation includes contributions from both uniform and post-uniform deformations.

The modest importance of carbon content (3.6%) despite its well-known effects on steel properties requires an explanation. Carbon influences formability through multiple pathways: strengthening the ferrite matrix through solid solution hardening, determining the carbon content of martensite (which controls martensite hardness), affecting the austenite-to-martensite transformation kinetics, and influencing carbide precipitation behavior [51]. However, once the microstructure (phase fractions) and mechanical properties (UTS and n-value) are specified, the carbon content provides limited additional predictive value because its effects are indirectly captured through these other features. This finding suggests that direct compositional specifications may be less important than the resulting microstructure and properties when predicting formability, which has practical implications for alloy-design strategies.

### 4.3. Comparison with Empirical Prediction Methods

Traditional approaches to HER prediction rely on simple empirical correlations, typically relating the HER to a single mechanical property such as the ultimate tensile strength or hardness [52]. These correlations take the form HER = a + b × UTS or HER = c × exp(−d × UTS), where the coefficients are fitted to limited experimental datasets. Although simple correlations provide rough estimates, they suffer from several limitations that the present machine-learning approach addresses. First, single-variable correlations cannot capture the complex interactions between composition, microstructure, and properties. The machine learning model explicitly accounts for coupled effects, where, for example, the impact of the martensite fraction on the HER depends on the simultaneous carbon content and overall strength level. Second, empirical correlations trained on narrow datasets generalize poorly for steel outside the training range. The 300-sample synthetic dataset spanned a broad design space, enabling the machine-learning model to interpolate reliably across diverse conditions. Third, traditional correlations provide no mechanism for quantifying feature importance or guiding alloy design priorities. Feature importance analysis identifies the factors that merit the most attention during the development phase.

Comparing the predictions quantitatively, a simple linear regression of HER vs. UTS achieved R^2^ = 0.511 in the validation set, explaining approximately 51% of the variance. This represents the baseline performance of a simple model. The optimized Gradient Boosting model’s R^2^ = 0.796 explains an additional 28.5 pp of variance, representing a 56% relative improvement in the explained variance. This improvement is attributed to the capture of nonlinear relationships (strength-dependent regime change at 700 MPa UTS), interaction effects (interplay between phase fractions and mechanical properties), and multivariate patterns (combined influence of multiple composition and microstructure features). The RMSE improvement from linear regression (6.45%) to Gradient Boosting (5.81%) translates to a 10% reduction in the typical prediction error, which could enable more aggressive material specifications or reduce safety factors in automotive component design.

However, the machine learning approach introduces trade-offs. The Gradient Boosting model with 100 trees and 14 input features is substantially more complex than a two-parameter linear correlation, making it less transparent and harder to interpret without computational tools. Implementing the model requires Python with Scikit-learn or equivalent machine learning frameworks, whereas empirical equations can be evaluated using spreadsheets or simple calculators. The predictions of machine learning models lack smooth, continuous derivatives that facilitate optimization algorithms for alloy design; however, this limitation can be addressed using surrogate modeling techniques [53]. Perhaps most importantly, the reliability of machine learning models depends critically on the quality of the training data, whether experimental or synthetic, whereas empirical correlations based on physical principles maintain their validity even with limited calibration data.

### 4.4. Limitations and Considerations

This study demonstrates that physics-informed synthetic data can produce machine learning models with predictive accuracy (R^2^ = 0.80, RMSE = 5.8%) approaching the experimental-data-trained models reported in the literature (R^2^ = 0.85) [20]. However, several limitations define the appropriate application boundaries. The test set performance of the model reflects generalization within the synthetic data distribution but does not guarantee equivalent accuracy for real steel. Real materials introduce complexities that are not explicitly modeled, such as microstructural banding, crystallographic texture, non-metallic inclusions, edge condition variations from punch wear, and residual stresses from processing. These factors could increase the prediction errors to 8–12% RMSE and introduce systematic biases for certain material classes. Experimental validation using 30–50 carefully selected steels spanning the DP590-DP1180 compositional space remains a critical next step to quantify real-world accuracy.

The model was calibrated for conventional dual-phase steels with ferrite-bainite-martensite microstructures (C: 0.05–0.20%, Mn: 0.8–2.7%, UTS: 600–1150 MPa) tested according to the ISO 16630 standard (sheared edges, room temperature, quasi-static loading). Extrapolation beyond these ranges or application to other AHSS families, such as TRIP steels with retained austenite [54], Q&P steels with carbon partitioning [55], or medium-Mn steels with ultrafine grains [56], requires modified generation algorithms. The model does not account for edge preparation variations (machined vs. laser-cut), testing conditions (temperature and strain rate), or processing parameters (annealing cycles and cooling rates).

Synthetic data generation involves human judgment in selecting empirical relationships, calibrating coefficients (α_VB_ = 20, α_VM_ = 18), and choosing which phenomena to model. If the selected relationships are incomplete or valid only under specific conditions, the predictions inherit these limitations. We mitigated this through multi-source validation and confirmed that the feature importance rankings matched independent experimental findings [20], but ultimate validation requires direct experimental comparison. This approach enables 50–80% experimental burden reduction by screening 100 candidates to 10–20 focused targets requiring physical testing. Appropriate applications include preliminary design space exploration, hypothesis generation, and educational demonstration. Inappropriate applications without experimental validation include safety-critical component certification, direct production implementation and regulatory submissions. Organizations deploying such models should implement phased validation (synthetic screening → experimental testing → pilot production), input domain checking for extrapolations, and expert reviews by metallurgists.

The 300-sample dataset, although larger than typical academic studies, remains modest by machine learning standards. The Gradient Boosting architecture may represent a performance ceiling without substantially larger training sets. The 3-fold cross-validation provided adequate algorithm comparison but less robust performance estimates than the 5-fold or 10-fold alternatives, justified by computational constraints and validated by independent test set evaluation. These reflect responsible scientific communication and define appropriate application boundaries rather than indicating methodological inadequacy. The substantial positive results (R^2^ = 0.80, physically meaningful feature rankings, cost-effective generation) demonstrate that physics-informed synthetic data successfully complement experimental research when applied within proper constraints.

### 4.5. Practical Implications and Future Directions

Despite these limitations, the validated model enables several practical applications in the development of advanced high-strength steels. First, rapid virtual screening can evaluate candidate compositions and microstructures without expensive experimental testing. A typical alloy development program might consider 50–100 composition variations; predicting the HER computationally reduces the number of physical tests needed from dozens to approximately 10–20 focused validation experiments. This accelerates development cycles from months to weeks while reducing material and testing costs by 50–80%. Second, the model supports multi-objective optimization, where automotive engineers must balance the strength, elongation, hole expansion ratio, crash energy absorption, and cost [57]. Combining the HER prediction model with equivalent models for other properties enables Pareto frontier analysis to identify optimal trade-offs [58]. Third, the feature importance rankings guide R&D priorities by quantifying which factors deserve the most attention. The finding that phase balance (VB, VM) is nearly as important as overall strength (UTS) suggests that microstructure control through heat-treatment optimization deserves investment comparable to compositional development. Fourth, the model provides a foundation for inverse design approaches, wherein engineers specify the desired property targets, and computational algorithms identify the compositions and processing routes likely to achieve those targets [59]. Traditional trial-and-error experimentation progresses from composition to properties, requiring many iterations to identify satisfactory combinations. Inverse design reverses this flow, dramatically accelerating the discovery of materials that meet the application requirements. Fifth, the transparent synthetic data generation methodology enables technology transfer and collaborative development. Other researchers can reproduce the exact dataset, generate variations by exploring different assumptions or validate the predictions against their experimental databases. This openness contrasts with proprietary experimental data, which remain locked within individual companies and research groups.

Future studies should prioritize several directions to build on these findings. The most immediate experimental validation using carefully designed test matrices spanning the composition and microstructure spaces would quantify the real-world prediction accuracy. Collaboration with steel producers or automotive companies could provide access to production data for the validation. Transfer learning approaches can fine-tune models using limited experimental data while retaining the benefits of large-scale synthetic training [60]. Expanding the synthetic data generation framework to include processing parameters (intercritical annealing temperature, cooling rate, and coiling temperature) would enable the prediction of as-processed properties, rather than requiring microstructural characterization. Incorporating edge condition variables (shear clearance, punch wear, and cutting method) would make the predictions more relevant to the manufacturing conditions. Extending to additional AHSS families (TRIP, Q&P, and medium Mn) would broaden the applicability across the automotive materials portfolio.

From a methodological perspective, exploring alternative machine learning architectures could help identify performance improvement. Ensemble methods that combine Gradient Boosting with neural networks may capture complementary patterns [61]. Physics-informed neural networks with loss functions that encode known constraints can improve generalization [62]. Gaussian process models would provide prediction uncertainty estimates that are crucial for risk-aware decision-making [63]. Graphs that treat composition-microstructure-property relationships as knowledge graphs can discover novel connections [64]. Automated machine learning (AutoML) frameworks can optimize the entire pipeline from feature engineering to algorithm selection and hyperparameter tuning [65,66]. The extension to welded joints represents a critical future direction for industrial deployment. In automotive body-in-white structures, dual-phase steels are predominantly joined by resistance spot welding and laser welding, creating heat-affected zones (HAZ) with altered microstructures and properties that influence formability. The HAZ in DP steels exhibits complex microstructural evolution: the subcritical HAZ undergoes martensite tempering (softening), the intercritical HAZ forms fresh martensite with grain refinement, and the supercritical HAZ develops coarse prior austenite grains that transform into hard martensite. These microstructural changes, combined with residual stresses from welding, can significantly affect edge formability. Developing synthetic data generation frameworks for weld formability prediction requires incorporating thermal cycle effects through Rosenthal heat flow solutions coupled with continuous cooling transformation (CCT) diagrams, HAZ width correlations as functions of heat input and thermal diffusivity, and residual stress distributions from thermal-mechanical finite element modeling. The machine learning architecture must accommodate spatial features and predict position-dependent HER as a function of the distance from the weld centerline. While computationally feasible, this extension would require experimental validation on actual spot-welded and laser-welded specimens with HER tests specifically targeting the HAZ regions, which is a substantial undertaking and represents the natural next step for the methodology established in the present work.

The broader vision is to establish physics-informed synthetic data generation as a standard methodology in materials informatics, complementing experimental research rather than replacing it. High-quality experimental data will always provide the gold standard for validation and the foundation for discovering new physical relationships. However, synthetic data offer powerful capabilities for rapid exploration, systematic hypothesis testing, and democratized access to machine learning tools when experimental data are scarce or proprietary. The key to success lies in the transparent documentation of the generation algorithms, rigorous validation of physical consistency, and honest discussion of limitations and uncertainties. As demonstrated here, when these principles are followed, synthetic data can produce machine learning models with performance approaching those trained on experimental measurements while requiring a small fraction of the resources.

## 5. Conclusions

This study developed and validated a machine learning framework for predicting the hole expansion ratio in advanced high-strength steels using physics-informed synthetic data. The key findings and contributions of this study are as follows.

The optimized Gradient Boosting model achieved excellent predictive performance on an independent test set (R^2^ = 0.796, RMSE = 5.81%, MAE = 4.93%), demonstrating that machine learning models trained on carefully constructed synthetic data can approach the accuracy of models trained on experimental measurements. The prediction error of approximately 5.8% fell within the typical experimental scatter range for the ISO 16630-hole expansion testing, suggesting its practical utility for industrial applications.

Feature importance analysis revealed physically meaningful rankings that validated our synthetic data approach. The ultimate tensile strength dominated with 40.9% importance, confirming the fundamental strength–formability trade-off documented over decades of AHSS research. The volume fractions of bainite (15.1%) and martensite (14.7%) were nearly equal, indicating that phase balance optimization is crucial for achieving superior edge formability. The strain hardening exponent ranked fourth (12.4%), reflecting its role in controlling the plastic instability and strain distribution. The top four features collectively accounted for 83.1% of the predictive power, suggesting that parsimonious models focusing on these dominant factors could achieve near-optimal performance.

The physics-informed synthetic data generation methodology successfully encoded validated empirical relationships from peer-reviewed literature [20,33,34], including Chen et al.’s strength-dependent HER behavior, microstructural effects, and tensile property correlations. The resulting 300-sample dataset exhibited realistic feature distributions that matched the commercial AHSS specifications and internally consistent correlations that aligned with the metallurgical principles. The inclusion of appropriate experimental noise (5% standard deviation) prevented overfitting while maintaining physical fidelity.

A comparison with baseline models demonstrated that sophisticated ensemble methods substantially outperformed simple linear correlations, explaining an additional 28.5% of the variance beyond the single-variable models. The 10% reduction in the typical prediction error (from 6.45% to 5.81% RMSE) could enable more aggressive material specifications or reduced safety factors in automotive component design, translating to lighter vehicle structures and improved fuel efficiency. This study demonstrates a practical pathway for addressing the challenges of data scarcity in materials science informatics. Generating 300 synthetic samples required approximately one hour of computational time on standard hardware, whereas equivalent experimental testing would require months of laboratory work and substantial financial investment. This transparent methodology enables reproducibility and technology transfer, supporting open-science principles that accelerate collaborative material development.

This study provides a validated template for physics-informed synthetic data generation that can be adapted to other properties and material systems, thereby advancing machine learning applications in materials science. The key success factors include building generation algorithms exclusively on validated empirical relationships, ensuring realistic feature distributions that match commercial specifications, incorporating appropriate experimental noise, and rigorously validating physical consistency through feature importance analyses and correlation assessments. For industry practitioners developing advanced high-strength steels, the validated model enables rapid virtual screening of candidate compositions and microstructures, multi-objective optimization for balancing formability with other properties, and quantitative guidance on R&D priorities through feature-importance rankings. The model can reduce the experimental testing burden by 50–80% while accelerating the development cycles from months to weeks.

Future studies should prioritize experimental validation using carefully designed test matrices, transfer learning approaches combining synthetic training with limited experimental fine-tuning, expansion to include processing parameters and edge conditions, and extension to additional AHSS families, including TRIP, Q&P, and medium Mn steels. Exploring alternative machine learning architectures and establishing prediction uncertainty quantification can further enhance their practical utility.

## Figures and Tables

**Table 1:** Typical properties of commercial DP steel grades.

Grade	C (wt.%)	Mn (wt.%)	Si (wt.%)	VM (%)	VB (%)	VF (%)	YS (MPa)	UTS (MPa)	TE (%)	HER (%)	Reference
DP590	0.05–0.10	1.0–1.2	0.3–0.6	20–30	5–15	60–70	340–400	590–670	23–27	50–70	[35]
DP780	0.09–0.10	1.6–2.1	0.3	30–40	10–20	45–55	420–520	750–850	17–20	35–50	[35]
DP980	0.09–0.15	1.4–2.2	0.3–0.65	45–55	10–20	30–40	550–650	980–1080	12–16	25–40	[35]
DP1180	0.13–0.18	1.8–2.5	0.4–0.7	55–70	5–15	20–30	800–900	1150–1250	9–12	20–30	[36]

Note: The properties represent typical values from commercial specifications; actual properties vary with specific processing conditions.

**Table 2:** Total elongation ranges by strength level.

UTS Range (MPa)	Total Elongation (%)	Representative Grade
<650	22–30	DP590
650–850	16–22	DP780
850–1050	12–16	DP980
>1050	8–12	DP1180

**Table 3:** Baseline model performance on validation set (n = 60).

Model	R^2^	RMSE (%)	MAE (%)
AdaBoost	0.64	5.52	4.63
Extra Trees	0.60	5.85	4.65
SVR (Linear)	0.54	6.26	4.90
Lasso	0.52	6.38	5.07
Random Forest	0.52	6.41	5.06
Linear Regression	0.51	6.45	5.18
ElasticNet	0.51	6.48	5.12
Ridge	0.50	6.49	5.08
Gradient Boosting	0.45	6.83	5.02
K-Nearest Neighbors	0.42	7.04	5.47
SVR (RBF)	0.34	7.50	5.64
Decision Tree	0.20	8.27	6.46

**Table 4:** Optimized hyperparameters and cross-validation performance.

Model	Best Hyperparameters	CV R^2^
Gradient Boosting	n_estimators_ = 100, max_depth = 3, learning_rate = 0.05, subsample = 0.8, min_samples_split = 2	0.61
Random Forest	n_estimators_ = 200, max_depth = 20, max_features = ‘sqrt’, min_samples_leaf = 1, min_samples_split = 2	0.60
Extra Trees	n_estimators_ = 100, max_depth = None, max_features = ‘sqrt’, min_samples_leaf = 1, min_samples_split = 2	0.59
SVR	C = 10, ϵ = 0.1, γ = 0.01	0.50

**Table 5:** Test set performance of optimized models (n = 60).

Model	R^2^	RMSE (%)	MAE (%)
Gradient Boosting (Tuned)	0.80	5.81	4.93
Random Forest (Tuned)	0.74	6.52	5.33
Extra Trees (Tuned)	0.68	7.22	5.63
SVR (Tuned)	0.45	9.50	6.64

**Table 6:** Feature importance rankings from Gradient Boosting model.

Rank	Feature	Importance	Physical Significance
1	UTS	0.409	Ultimate tensile strength—inverse strength–formability relationship
2	VB	0.151	Bainite volume fraction—promotes ductility and toughness
3	VM	0.147	Martensite volume fraction—increases strength but reduces ductility
4	n	0.124	Strain hardening exponent—controls plastic instability
5	C	0.036	Carbon content—solid solution and phase transformation effects
6	TE	0.033	Total elongation—indicator of bulk ductility
7	YS	0.017	Yield strength—baseline strength level
8	Mo	0.016	Molybdenum—minor alloying addition
9	Si	0.015	Silicon—solid solution strengthening
10	Mn	0.014	Manganese—austenite stabilizer
11	r	0.012	Plastic strain ratio—anisotropy indicator
12	CE	0.010	Carbon equivalent—hardenability metric
13	VF	0.009	Ferrite volume fraction—soft ductile phase
14	Cr	0.007	Chromium—hardenability element

## Data Availability

The original contributions presented in this study are included in the article and Supplementary Material. Further inquiries can be directed to the corresponding authors.

## Supplementary Materials

The following supporting information can be downloaded at: https://www.mdpi.com/article/10.3390/ma19081592/s1.

## Abbreviations

Abbreviation	Full Term
AHSS	Advanced High-Strength Steel
DP	Dual Phase
HER	Hole Expansion Ratio
UTS	Ultimate Tensile Strength
YS	Yield Strength
TE	Total Elongation
VM	Martensite Volume Fraction (%)
VB	Bainite Volume Fraction (%)
VF	Ferrite Volume Fraction (%)
C	Carbon Content (wt.%)
Mn	Manganese Content (wt.%)
Si	Silicon Content (wt.%)
Cr	Chromium Content (wt.%)
Mo	Molybdenum Content (wt.%)
CE	Carbon Equivalent
n	Strain Hardening Exponent
r	Plastic Strain Ratio
ML	Machine Learning
RF	Random Forest
ET	Extra Trees
GB	Gradient Boosting
SVR	Support Vector Regression
CV	Cross-Validation
RMSE	Root Mean Square Error
MAE	Mean Absolute Error
R^2^	Coefficient of Determination
