# Supplementary material for: Prediction of Hole Expansion Ratio in Advanced High-Strength Steels Using Physics-Informed Machine Learning
Source: Materials (Basel). 2026 Apr 15;19(8):1592. doi: 10.3390/ma19081592 (PMC13117724; doi:10.3390/ma19081592)
Supplement: Supplementary file 1 [file materials-19-01592-s001.zip › materials-4052346-supplementary.pdf]

File S1

| id | C | Mn | Si | Cr | Mo | CE | VF | VB | VM | YS | UTS | TE | n | r | HER |
|---|---|---|---|---|---|---|---|---|---|---|---|---|---|---|---|
| 1 | 0.056 | 2.38 | 0.46 | 0.46 | 0.049 | 0.647 | 18.2 | 47.6 | 34.2 | 478 | 804 | 16.2 | 0.124 | 0.92 | 42.1 |
| 2 | 0.137 | 2.28 | 0.2 | 0.46 | 0.14 | 0.678 | 38.8 | 43.6 | 17.6 | 514 | 751 | 20.1 | 0.135 | 1.03 | 48.4 |
| 3 | 0.062 | 1.65 | 0.22 | 0.49 | 0.007 | 0.48 | 43 | 36.4 | 20.6 | 433 | 678 | 20.1 | 0.142 | 1.03 | 62.9 |
| 4 | 0.17 | 1.54 | 0.37 | 0.12 | 0.122 | 0.551 | 33.8 | 33.9 | 32.3 | 584 | 884 | 12.6 | 0.11 | 0.72 | 33.6 |
| 5 | 0.177 | 1.02 | 0.14 | 0.46 | 0.001 | 0.467 | 54.6 | 14.8 | 30.6 | 458 | 803 | 18 | 0.127 | 1.13 | 26.1 |
| 6 | 0.181 | 1.41 | 0.48 | 0.28 | 0.044 | 0.578 | 22.9 | 53.8 | 23.3 | 627 | 846 | 21.6 | 0.128 | 0.76 | 44.8 |
| 7 | 0.165 | 1.35 | 0.44 | 0.16 | 0.06 | 0.522 | 30.1 | 20.9 | 48.9 | 644 | 996 | 15.7 | 0.104 | 1.3 | 39.3 |
| 8 | 0.179 | 2.65 | 0.37 | 0.16 | 0.192 | 0.766 | 38.2 | 23 | 38.7 | 636 | 875 | 12.2 | 0.111 | 1.11 | 25.2 |
| 9 | 0.069 | 1.49 | 0.25 | 0.15 | 0.073 | 0.411 | 39.2 | 5.1 | 55.7 | 492 | 891 | 15.7 | 0.114 | 0.74 | 34.8 |
| 10 | 0.118 | 1.04 | 0.28 | 0.45 | 0.19 | 0.476 | 50.2 | 33.6 | 16.2 | 459 | 690 | 17.1 | 0.137 | 1.39 | 65.6 |
| 11 | 0.055 | 1.22 | 0.52 | 0.22 | 0.067 | 0.419 | 16.1 | 5.5 | 78.4 | 620 | 1041 | 12.8 | 0.095 | 1.32 | 23.3 |
| 12 | 0.109 | 2.22 | 0.65 | 0.3 | 0.151 | 0.701 | 29.4 | 18.4 | 52.2 | 618 | 891 | 12.5 | 0.11 | 1.35 | 31.1 |
| 13 | 0.137 | 2.2 | 0.2 | 0.01 | 0.095 | 0.564 | 17.8 | 22 | 60.2 | 643 | 1028 | 15.3 | 0.1 | 0.73 | 28.8 |
| 14 | 0.107 | 1.19 | 0.51 | 0.28 | 0.094 | 0.481 | 48.2 | 16.3 | 35.5 | 510 | 825 | 21.1 | 0.129 | 1.15 | 37.5 |
| 15 | 0.121 | 2.46 | 0.36 | 0.29 | 0.002 | 0.661 | 68.8 | 12.4 | 18.7 | 446 | 696 | 18.9 | 0.139 | 0.97 | 56.9 |
| 16 | 0.058 | 1.63 | 0.46 | 0.45 | 0.008 | 0.513 | 55.8 | 25 | 19.2 | 443 | 645 | 28.7 | 0.159 | 1.05 | 76.4 |
| 17 | 0.193 | 1.54 | 0.1 | 0.45 | 0.165 | 0.594 | 18.2 | 11.3 | 70.5 | 763 | 1118 | 9.2 | 0.082 | 0.92 | 19.8 |
| 18 | 0.12 | 1.89 | 0.38 | 0.29 | 0.032 | 0.574 | 26.8 | 2.2 | 71 | 634 | 1033 | 14.2 | 0.098 | 0.83 | 18.2 |
| 19 | 0.07 | 1.06 | 0.67 | 0.17 | 0.154 | 0.446 | 18.2 | 15.9 | 65.9 | 633 | 966 | 12.6 | 0.102 | 0.94 | 32.9 |
| 20 | 0.194 | 2.33 | 0.55 | 0.3 | 0.044 | 0.761 | 28.2 | 27.6 | 44.2 | 520 | 932 | 13.6 | 0.107 | 0.94 | 22.3 |
| 21 | 0.161 | 1.85 | 0.31 | 0.37 | 0.031 | 0.611 | 26.3 | 10.1 | 63.6 | 634 | 1042 | 13.7 | 0.096 | 1.25 | 24.5 |
| 22 | 0.052 | 2.17 | 0.11 | 0.43 | 0.092 | 0.539 | 45.1 | 31.1 | 23.9 | 412 | 720 | 21.9 | 0.141 | 1.25 | 46 |
| 23 | 0.097 | 2.32 | 0.15 | 0.28 | 0.155 | 0.6 | 25.1 | 3.6 | 71.3 | 588 | 1003 | 13.1 | 0.099 | 1.19 | 15.5 |
| 24 | 0.185 | 1.47 | 0.49 | 0.44 | 0.019 | 0.618 | 19 | 14.5 | 66.5 | 681 | 1049 | 13.4 | 0.095 | 0.92 | 22.8 |
| 25 | 0.162 | 0.81 | 0.48 | 0.24 | 0.111 | 0.463 | 23.7 | 37.5 | 38.8 | 565 | 908 | 12.4 | 0.108 | 1.37 | 37.6 |
| 26 | 0.087 | 1.19 | 0.64 | 0.02 | 0.092 | 0.436 | 45.6 | 18.4 | 36 | 505 | 820 | 19.6 | 0.127 | 1.09 | 34.1 |
| 27 | 0.11 | 1.86 | 0.55 | 0.16 | 0.121 | 0.586 | 27.4 | 12 | 60.7 | 614 | 947 | 14.6 | 0.107 | 0.97 | 29.1 |
| 28 | 0.088 | 2.06 | 0.55 | 0.44 | 0.044 | 0.637 | 28.2 | 41.6 | 30.2 | 468 | 805 | 16.3 | 0.124 | 1.01 | 37 |
| 29 | 0.078 | 2.67 | 0.39 | 0.4 | 0.019 | 0.684 | 27.4 | 1.5 | 71.2 | 683 | 949 | 15.9 | 0.109 | 0.91 | 28.7 |
| 30 | 0.167 | 2.02 | 0.15 | 0.1 | 0.132 | 0.58 | 26.1 | 1.4 | 72.5 | 708 | 1095 | 10.2 | 0.086 | 1.29 | 17.2 |
| 31 | 0.085 | 1.38 | 0.36 | 0.43 | 0.066 | 0.486 | 52.2 | 31.9 | 15.9 | 408 | 671 | 21.1 | 0.144 | 1.28 | 65 |
| 32 | 0.153 | 0.86 | 0.61 | 0.43 | 0.171 | 0.539 | 29.9 | 39.7 | 30.4 | 516 | 860 | 13.2 | 0.114 | 1.39 | 27.2 |
| 33 | 0.12 | 1.62 | 0.53 | 0.19 | 0.161 | 0.566 | 66.9 | 12.6 | 20.5 | 404 | 673 | 19.6 | 0.142 | 1.19 | 62.4 |
| 34 | 0.066 | 1.53 | 0.66 | 0.4 | 0.159 | 0.564 | 27.1 | 0.2 | 72.6 | 736 | 988 | 15.4 | 0.104 | 1.15 | 31.7 |
| 35 | 0.196 | 2.49 | 0.41 | 0.45 | 0.002 | 0.783 | 46 | 23.6 | 30.4 | 651 | 905 | 14.8 | 0.112 | 0.72 | 29.5 |
| 36 | 0.074 | 2.13 | 0.54 | 0.35 | 0.148 | 0.638 | 51.2 | 10.3 | 38.5 | 517 | 777 | 19.3 | 0.131 | 1.06 | 39.3 |
| 37 | 0.128 | 1.27 | 0.19 | 0.1 | 0.159 | 0.429 | 18.4 | 17 | 64.5 | 601 | 1055 | 9.9 | 0.089 | 1.12 | 22.8 |
| 38 | 0.186 | 1.76 | 0.29 | 0.21 | 0.15 | 0.61 | 21.9 | 53.1 | 25 | 602 | 905 | 13.5 | 0.11 | 1.09 | 32.9 |
| 39 | 0.138 | 1.25 | 0.19 | 0.5 | 0.094 | 0.503 | 28.8 | 2.9 | 68.4 | 629 | 1017 | 15 | 0.101 | 0.72 | 19.3 |
| 40 | 0.073 | 1.9 | 0.1 | 0.34 | 0.09 | 0.496 | 28.3 | 28.4 | 43.3 | 499 | 824 | 19 | 0.126 | 1.32 | 40.7 |
| 41 | 0.1 | 1.48 | 0.43 | 0.27 | 0.05 | 0.496 | 29 | 0 | 71 | 557 | 994 | 13.8 | 0.101 | 0.79 | 21.1 |
| 42 | 0.106 | 1.02 | 0.39 | 0.32 | 0.088 | 0.435 | 30.1 | 10.6 | 59.4 | 549 | 951 | 14 | 0.106 | 1.2 | 31.4 |
| 43 | 0.175 | 0.95 | 0.23 | 0.1 | 0.042 | 0.408 | 34.6 | 8 | 57.4 | 776 | 1037 | 15.3 | 0.099 | 0.97 | 19.6 |

| | | | | | | | | | | | | | | | |
|---|---|---|---|---|---|---|---|---|---|---|---|---|---|---|---|
| 44 | 0.109 | 2.25 | 0.67 | 0.17 | 0.175 | 0.685 | 33.8 | 2.6 | 63.6 | 533 | 958 | 15.8 | 0.108 | 1.12 | 16.9 |
| 45 | 0.133 | 0.88 | 0.42 | 0.1 | 0.007 | 0.385 | 24.4 | 57.3 | 18.2 | 451 | 802 | 18.3 | 0.127 | 0.87 | 40.4 |
| 46 | 0.172 | 1.42 | 0.2 | 0.08 | 0.094 | 0.484 | 76.6 | 0.7 | 22.8 | 409 | 716 | 16.9 | 0.134 | 1.22 | 29.2 |
| 47 | 0.063 | 0.98 | 0.2 | 0.29 | 0.181 | 0.361 | 24.1 | 0.5 | 75.4 | 621 | 955 | 15.3 | 0.107 | 1.38 | 17.2 |
| 48 | 0.093 | 0.8 | 0.58 | 0.1 | 0.155 | 0.393 | 23.2 | 9.4 | 67.4 | 636 | 1025 | 14.4 | 0.099 | 1.29 | 22.4 |
| 49 | 0.166 | 2.16 | 0.18 | 0.29 | 0.13 | 0.646 | 32.7 | 23.8 | 43.6 | 645 | 957 | 13.7 | 0.105 | 1.29 | 30.7 |
| 50 | 0.088 | 1.58 | 0.57 | 0.28 | 0.008 | 0.523 | 23.7 | 37.2 | 39 | 518 | 827 | 20.7 | 0.128 | 1.03 | 29 |
| 51 | 0.187 | 0.83 | 0.18 | 0.45 | 0.109 | 0.474 | 24.4 | 33.1 | 42.5 | 628 | 931 | 14.3 | 0.108 | 1.28 | 33.6 |
| 52 | 0.119 | 2.45 | 0.57 | 0.43 | 0.046 | 0.736 | 23.1 | 2.3 | 74.5 | 578 | 1024 | 13.6 | 0.098 | 0.91 | 24.4 |
| 53 | 0.193 | 2.45 | 0.51 | 0.25 | 0.039 | 0.762 | 39 | 16.5 | 44.5 | 558 | 957 | 12.3 | 0.103 | 1.35 | 23 |
| 54 | 0.065 | 1.77 | 0.28 | 0.37 | 0.101 | 0.508 | 58.7 | 2 | 39.3 | 425 | 733 | 18.1 | 0.134 | 1.16 | 40 |
| 55 | 0.077 | 2.14 | 0.21 | 0.03 | 0.2 | 0.521 | 34.3 | 0.3 | 65.4 | 697 | 967 | 12.5 | 0.102 | 0.82 | 35.2 |
| 56 | 0.161 | 2.47 | 0.4 | 0.21 | 0.038 | 0.702 | 24.4 | 41.1 | 34.5 | 613 | 883 | 13.6 | 0.112 | 1.09 | 34.7 |
| 57 | 0.09 | 1.43 | 0.44 | 0.28 | 0.185 | 0.51 | 15.9 | 23.7 | 60.4 | 580 | 963 | 12.4 | 0.102 | 1.39 | 24 |
| 58 | 0.199 | 1.82 | 0.7 | 0.03 | 0.086 | 0.664 | 38.8 | 36.2 | 25 | 566 | 846 | 19 | 0.124 | 1.27 | 41.2 |
| 59 | 0.146 | 0.82 | 0.62 | 0.27 | 0.091 | 0.48 | 33.1 | 13 | 53.9 | 591 | 924 | 12.8 | 0.107 | 1.17 | 27.9 |
| 60 | 0.16 | 1.48 | 0.17 | 0.22 | 0.04 | 0.493 | 28.4 | 14.7 | 56.9 | 603 | 992 | 13.7 | 0.101 | 0.89 | 26.2 |
| 61 | 0.169 | 1.98 | 0.32 | 0.11 | 0.042 | 0.594 | 16.6 | 18.8 | 64.6 | 618 | 1102 | 9 | 0.083 | 0.99 | 23.5 |
| 62 | 0.195 | 2.13 | 0.19 | 0.21 | 0.096 | 0.648 | 55.9 | 0.4 | 43.7 | 631 | 882 | 13.7 | 0.112 | 1.24 | 27.1 |
| 63 | 0.168 | 1.18 | 0.39 | 0.01 | 0.176 | 0.479 | 52.2 | 27.6 | 20.2 | 581 | 802 | 20.9 | 0.131 | 0.85 | 39.8 |
| 64 | 0.085 | 1.55 | 0.2 | 0.01 | 0.18 | 0.422 | 48.3 | 33.7 | 18 | 460 | 672 | 19 | 0.141 | 0.73 | 65.7 |
| 65 | 0.07 | 1.19 | 0.25 | 0.06 | 0.063 | 0.342 | 54.6 | 17 | 28.4 | 530 | 715 | 16.1 | 0.133 | 1.02 | 33.5 |
| 66 | 0.138 | 2.21 | 0.32 | 0.35 | 0.176 | 0.675 | 18.6 | 16.7 | 64.7 | 698 | 1013 | 12.3 | 0.097 | 1.04 | 24.7 |
| 67 | 0.073 | 1.52 | 0.49 | 0.16 | 0.188 | 0.496 | 34 | 5.4 | 60.6 | 643 | 877 | 15.5 | 0.116 | 0.93 | 25.6 |
| 68 | 0.19 | 2.67 | 0.33 | 0.17 | 0.008 | 0.735 | 31.3 | 16.6 | 52.1 | 620 | 990 | 13.1 | 0.101 | 0.84 | 26.8 |
| 69 | 0.087 | 1.83 | 0.19 | 0.01 | 0.072 | 0.447 | 43.2 | 31.8 | 25 | 476 | 722 | 19.3 | 0.137 | 0.92 | 35.8 |
| 70 | 0.079 | 1.03 | 0.5 | 0.27 | 0.083 | 0.42 | 39.8 | 12.8 | 47.4 | 505 | 844 | 16.5 | 0.12 | 1.37 | 34.6 |
| 71 | 0.149 | 1.85 | 0.15 | 0.22 | 0.036 | 0.54 | 38.8 | 14.3 | 47 | 643 | 914 | 16 | 0.113 | 1.14 | 27.3 |
| 72 | 0.158 | 1.57 | 0.38 | 0.11 | 0.117 | 0.541 | 39.8 | 30.7 | 29.5 | 545 | 852 | 13.4 | 0.115 | 1.38 | 38 |
| 73 | 0.194 | 1.65 | 0.63 | 0.05 | 0.083 | 0.621 | 69.9 | 14 | 16.1 | 523 | 717 | 16.5 | 0.133 | 1.38 | 33.5 |
| 74 | 0.092 | 1.73 | 0.52 | 0.36 | 0.025 | 0.56 | 21.5 | 30.1 | 48.4 | 506 | 878 | 12.8 | 0.111 | 1.39 | 37.6 |
| 75 | 0.066 | 0.88 | 0.51 | 0.3 | 0.074 | 0.39 | 16.5 | 4 | 79.5 | 693 | 1030 | 14.3 | 0.098 | 0.73 | 31.8 |
| 76 | 0.105 | 1.05 | 0.32 | 0.45 | 0.114 | 0.458 | 18.1 | 56.9 | 24.9 | 474 | 810 | 17.6 | 0.125 | 1.26 | 41.5 |
| 77 | 0.184 | 2.34 | 0.38 | 0.16 | 0.14 | 0.71 | 16 | 12.2 | 71.8 | 641 | 1149 | 10.7 | 0.081 | 1.29 | 21.1 |
| 78 | 0.058 | 2.5 | 0.47 | 0.03 | 0.018 | 0.579 | 17 | 7.4 | 75.6 | 619 | 974 | 15.8 | 0.106 | 1.23 | 28.2 |
| 79 | 0.096 | 1.97 | 0.53 | 0.11 | 0.024 | 0.557 | 32.1 | 9.6 | 58.3 | 537 | 963 | 14.1 | 0.105 | 0.95 | 24.6 |
| 80 | 0.158 | 2.53 | 0.69 | 0.26 | 0.057 | 0.781 | 41 | 37.9 | 21 | 613 | 824 | 16.1 | 0.122 | 1.19 | 44.1 |
| 81 | 0.126 | 2.09 | 0.52 | 0.29 | 0.026 | 0.642 | 59.7 | 13.3 | 26.9 | 442 | 779 | 18.8 | 0.13 | 1.26 | 30.9 |
| 82 | 0.081 | 1.75 | 0.55 | 0.27 | 0.053 | 0.548 | 20.1 | 11.6 | 68.3 | 671 | 1021 | 14.6 | 0.1 | 0.79 | 28.5 |
| 83 | 0.157 | 1.21 | 0.32 | 0.36 | 0.038 | 0.503 | 18 | 8.8 | 73.2 | 809 | 1124 | 10.9 | 0.084 | 0.88 | 29.7 |
| 84 | 0.094 | 1.52 | 0.52 | 0.46 | 0.107 | 0.564 | 37.6 | 14.1 | 48.3 | 646 | 866 | 12.2 | 0.112 | 0.82 | 32.5 |
| 85 | 0.125 | 1.95 | 0.48 | 0.37 | 0.129 | 0.647 | 24.9 | 30.9 | 44.2 | 536 | 925 | 15.2 | 0.11 | 0.7 | 30.5 |
| 86 | 0.198 | 2.13 | 0.39 | 0.4 | 0.007 | 0.712 | 15.2 | 52.3 | 32.5 | 610 | 961 | 13.3 | 0.104 | 0.88 | 37.7 |
| 87 | 0.165 | 0.87 | 0.43 | 0.15 | 0.111 | 0.448 | 16.8 | 6.2 | 77 | 724 | 1152 | 10.1 | 0.08 | 0.99 | 25.5 |

| | | | | | | | | | | | | | | | |
|---|---|---|---|---|---|---|---|---|---|---|---|---|---|---|---|
| 88 | 0.061 | 1.98 | 0.13 | 0.15 | 0.104 | 0.467 | 60.2 | 1 | 38.8 | 407 | 701 | 19.8 | 0.14 | 0.82 | 24 |
| 89 | 0.106 | 2.61 | 0.63 | 0.45 | 0.025 | 0.762 | 54.7 | 7.8 | 37.5 | 434 | 749 | 19.2 | 0.134 | 1.18 | 36.3 |
| 90 | 0.11 | 2.58 | 0.19 | 0.37 | 0.012 | 0.654 | 60.7 | 18.7 | 20.6 | 435 | 714 | 18.7 | 0.137 | 1.35 | 34 |
| 91 | 0.174 | 2.23 | 0.44 | 0.02 | 0.2 | 0.678 | 23.4 | 27.1 | 49.5 | 630 | 1016 | 12.6 | 0.097 | 0.71 | 31.3 |
| 92 | 0.107 | 1.46 | 0.5 | 0.35 | 0.15 | 0.548 | 23.2 | 47.8 | 29 | 517 | 862 | 13.3 | 0.114 | 1.18 | 49.5 |
| 93 | 0.157 | 1.97 | 0.49 | 0.18 | 0.194 | 0.658 | 29.1 | 1.5 | 69.4 | 766 | 1067 | 8.8 | 0.087 | 0.9 | 23.5 |
| 94 | 0.117 | 0.87 | 0.56 | 0.49 | 0.054 | 0.483 | 24 | 13.8 | 62.2 | 553 | 970 | 14.5 | 0.105 | 1.33 | 34.4 |
| 95 | 0.199 | 2.18 | 0.63 | 0.37 | 0.089 | 0.78 | 46.7 | 11.3 | 42 | 639 | 935 | 13.7 | 0.107 | 1.38 | 21.9 |
| 96 | 0.103 | 2.25 | 0.68 | 0.08 | 0.07 | 0.642 | 15.8 | 7.5 | 76.6 | 599 | 1066 | 8.9 | 0.087 | 1.36 | 30.1 |
| 97 | 0.159 | 1.85 | 0.44 | 0.33 | 0.183 | 0.658 | 27 | 24.8 | 48.3 | 643 | 959 | 12.7 | 0.103 | 0.88 | 25.3 |
| 98 | 0.128 | 1.53 | 0.63 | 0.44 | 0.161 | 0.629 | 40 | 32.5 | 27.5 | 494 | 766 | 16.1 | 0.128 | 1.04 | 38.1 |
| 99 | 0.181 | 2.41 | 0.57 | 0.11 | 0.184 | 0.755 | 22.3 | 10.9 | 66.8 | 589 | 1041 | 15.1 | 0.098 | 0.72 | 22.7 |
| 100 | 0.132 | 0.97 | 0.51 | 0.2 | 0.121 | 0.459 | 48.9 | 26.9 | 24.2 | 447 | 774 | 20.4 | 0.133 | 0.95 | 37.7 |
| 101 | 0.171 | 2.01 | 0.61 | 0.39 | 0.015 | 0.71 | 44.6 | 1.1 | 54.3 | 672 | 952 | 12.7 | 0.104 | 0.87 | 17.5 |
| 102 | 0.08 | 0.9 | 0.67 | 0.49 | 0.044 | 0.473 | 45.1 | 11.6 | 43.3 | 471 | 788 | 19.2 | 0.13 | 1.32 | 34.3 |
| 103 | 0.177 | 1.52 | 0.56 | 0.22 | 0.011 | 0.588 | 41.1 | 23.2 | 35.7 | 533 | 880 | 15.3 | 0.115 | 1.22 | 34.2 |
| 104 | 0.114 | 1.57 | 0.52 | 0.46 | 0.018 | 0.575 | 16.4 | 10.6 | 73.1 | 761 | 1022 | 14.3 | 0.099 | 1.18 | 17.6 |
| 105 | 0.171 | 1.31 | 0.67 | 0.5 | 0.151 | 0.653 | 61.3 | 5.3 | 33.4 | 558 | 830 | 17.4 | 0.123 | 1.4 | 26.8 |
| 106 | 0.169 | 2.14 | 0.15 | 0.14 | 0.171 | 0.617 | 72.6 | 2.4 | 25 | 522 | 720 | 19.8 | 0.138 | 0.81 | 32.5 |
| 107 | 0.189 | 1.48 | 0.27 | 0.13 | 0.104 | 0.537 | 54.6 | 2.2 | 43.1 | 539 | 912 | 14.4 | 0.11 | 1.27 | 20 |
| 108 | 0.191 | 1 | 0.25 | 0.07 | 0.079 | 0.436 | 76.5 | 1.1 | 22.4 | 440 | 746 | 20.7 | 0.136 | 0.92 | 33.4 |
| 109 | 0.199 | 2.27 | 0.2 | 0.18 | 0.19 | 0.691 | 79.4 | 1.8 | 18.7 | 544 | 755 | 18.5 | 0.132 | 0.75 | 33.5 |
| 110 | 0.195 | 1.36 | 0.26 | 0.07 | 0.002 | 0.488 | 22.8 | 17.5 | 59.7 | 665 | 1071 | 10.2 | 0.088 | 1.38 | 33.1 |
| 111 | 0.187 | 1.5 | 0.62 | 0.12 | 0.005 | 0.585 | 21.3 | 9.4 | 69.3 | 811 | 1084 | 9.7 | 0.086 | 1.29 | 18.8 |
| 112 | 0.155 | 1.25 | 0.61 | 0.35 | 0.004 | 0.556 | 22.9 | 0.4 | 76.7 | 624 | 1111 | 8.5 | 0.082 | 0.83 | 27.6 |
| 113 | 0.152 | 1.54 | 0.24 | 0.2 | 0.041 | 0.505 | 19.4 | 1.8 | 78.7 | 703 | 1137 | 8.4 | 0.079 | 0.77 | 23 |
| 114 | 0.087 | 2.56 | 0.15 | 0.3 | 0.146 | 0.633 | 37.2 | 33.5 | 29.4 | 529 | 813 | 20.1 | 0.129 | 1.1 | 29.8 |
| 115 | 0.17 | 2.22 | 0.61 | 0.25 | 0.034 | 0.72 | 46.2 | 31.2 | 22.6 | 477 | 793 | 18.6 | 0.129 | 0.75 | 41 |
| 116 | 0.136 | 1.01 | 0.18 | 0.09 | 0.071 | 0.374 | 18.6 | 9.1 | 72.4 | 769 | 1055 | 9.1 | 0.088 | 1.31 | 23.5 |
| 117 | 0.123 | 0.81 | 0.55 | 0.21 | 0.184 | 0.446 | 20.4 | 4.9 | 74.6 | 689 | 1037 | 13.6 | 0.097 | 0.85 | 31.4 |
| 118 | 0.08 | 2.08 | 0.22 | 0.26 | 0.02 | 0.526 | 35.4 | 28.6 | 36 | 481 | 803 | 18.7 | 0.128 | 1.34 | 35 |
| 119 | 0.173 | 1.83 | 0.67 | 0.12 | 0.025 | 0.641 | 31.5 | 35.4 | 33.1 | 627 | 868 | 13.9 | 0.114 | 1.32 | 34.5 |
| 120 | 0.143 | 2.3 | 0.7 | 0.01 | 0.089 | 0.686 | 20 | 14 | 66.1 | 688 | 1042 | 14.4 | 0.097 | 1.04 | 19 |
| 121 | 0.11 | 2.69 | 0.29 | 0.2 | 0.029 | 0.663 | 31.8 | 8.9 | 59.3 | 563 | 966 | 12.4 | 0.102 | 1.16 | 25.3 |
| 122 | 0.143 | 2.43 | 0.59 | 0.29 | 0.147 | 0.753 | 31.8 | 23 | 45.1 | 604 | 879 | 12.5 | 0.111 | 1.25 | 26.1 |
| 123 | 0.179 | 2.13 | 0.65 | 0.33 | 0.165 | 0.762 | 26.6 | 21.1 | 52.3 | 626 | 991 | 14.1 | 0.102 | 0.71 | 27 |
| 124 | 0.162 | 2.01 | 0.46 | 0.4 | 0.049 | 0.678 | 18.1 | 14.9 | 67 | 678 | 1064 | 8.8 | 0.087 | 0.95 | 22.7 |
| 125 | 0.112 | 1.9 | 0.31 | 0.4 | 0.046 | 0.58 | 18.2 | 1.8 | 80 | 765 | 1053 | 11.9 | 0.093 | 0.77 | 15 |
| 126 | 0.093 | 2.47 | 0.67 | 0.23 | 0.109 | 0.707 | 24.3 | 31.5 | 44.2 | 495 | 851 | 13.8 | 0.116 | 1.28 | 37.6 |
| 127 | 0.078 | 1.57 | 0.16 | 0.12 | 0.173 | 0.429 | 61.5 | 2.7 | 35.8 | 450 | 742 | 20.1 | 0.136 | 0.93 | 34.5 |
| 128 | 0.112 | 2.03 | 0.3 | 0.41 | 0.053 | 0.602 | 38.6 | 12.2 | 49.1 | 583 | 889 | 12.8 | 0.11 | 1.16 | 32.5 |
| 129 | 0.094 | 1.09 | 0.64 | 0.24 | 0.095 | 0.47 | 57.1 | 0.6 | 42.4 | 544 | 821 | 20.9 | 0.129 | 0.86 | 35.7 |
| 130 | 0.071 | 1.32 | 0.24 | 0.07 | 0.141 | 0.381 | 33.4 | 40.8 | 25.7 | 508 | 742 | 18.2 | 0.133 | 1.34 | 45.5 |
| 131 | 0.093 | 1.89 | 0.61 | 0.41 | 0.035 | 0.619 | 45.4 | 15.6 | 39 | 464 | 765 | 16.2 | 0.128 | 1.21 | 30.6 |

| | | | | | | | | | | | | | | | |
|---|---|---|---|---|---|---|---|---|---|---|---|---|---|---|---|
| 132 | 0.052 | 0.97 | 0.19 | 0.18 | 0.03 | 0.295 | 72.9 | 3.5 | 23.6 | 349 | 596 | 24.6 | 0.157 | 1.35 | 70.3 |
| 133 | 0.061 | 1.51 | 0.24 | 0.29 | 0.043 | 0.427 | 57.2 | 6 | 36.8 | 404 | 725 | 19.6 | 0.137 | 1.24 | 32.8 |
| 134 | 0.186 | 0.95 | 0.18 | 0.04 | 0.084 | 0.405 | 38.3 | 9.7 | 52 | 652 | 1011 | 14.5 | 0.101 | 1.06 | 25.1 |
| 135 | 0.165 | 2.24 | 0.4 | 0.25 | 0.102 | 0.689 | 65.5 | 1.5 | 33 | 589 | 832 | 21.5 | 0.129 | 1.04 | 27.5 |
| 136 | 0.103 | 0.8 | 0.47 | 0.28 | 0.088 | 0.403 | 22.5 | 4.2 | 73.3 | 721 | 1038 | 14.7 | 0.098 | 0.83 | 30.3 |
| 137 | 0.128 | 1.29 | 0.26 | 0.37 | 0.197 | 0.509 | 22.5 | 38.5 | 39.1 | 620 | 874 | 14 | 0.114 | 0.78 | 34.7 |
| 138 | 0.104 | 2.34 | 0.19 | 0.04 | 0.141 | 0.569 | 36.6 | 39 | 24.4 | 513 | 763 | 20.7 | 0.135 | 1.34 | 36.5 |
| 139 | 0.123 | 2 | 0.54 | 0.16 | 0.028 | 0.603 | 43.4 | 16.2 | 40.4 | 482 | 815 | 18.4 | 0.126 | 0.97 | 38.8 |
| 140 | 0.094 | 1.02 | 0.65 | 0.16 | 0.041 | 0.434 | 35.6 | 14.2 | 50.2 | 535 | 913 | 13.3 | 0.109 | 1.36 | 35.4 |
| 141 | 0.17 | 1.17 | 0.47 | 0.34 | 0.075 | 0.542 | 17.4 | 6.3 | 76.2 | 659 | 1116 | 8.7 | 0.082 | 1.2 | 19.2 |
| 142 | 0.132 | 1.67 | 0.2 | 0.45 | 0.095 | 0.559 | 32 | 16.7 | 51.3 | 668 | 944 | 13.8 | 0.106 | 1.21 | 25.7 |
| 143 | 0.185 | 1.83 | 0.25 | 0.11 | 0.033 | 0.568 | 15.7 | 12.5 | 71.9 | 644 | 1120 | 11.5 | 0.085 | 0.95 | 26.5 |
| 144 | 0.116 | 2.67 | 0.53 | 0.12 | 0.15 | 0.721 | 48.2 | 10.5 | 41.2 | 522 | 843 | 17.7 | 0.122 | 1.19 | 35.5 |
| 145 | 0.066 | 1.31 | 0.66 | 0.31 | 0.021 | 0.482 | 19 | 3.8 | 77.3 | 711 | 1016 | 14.4 | 0.1 | 1.22 | 34.1 |
| 146 | 0.061 | 2.63 | 0.12 | 0.09 | 0.011 | 0.545 | 19.6 | 7.5 | 72.9 | 700 | 976 | 13.4 | 0.102 | 1.06 | 28.6 |
| 147 | 0.152 | 1.47 | 0.63 | 0.31 | 0.087 | 0.6 | 16.4 | 44.7 | 38.9 | 561 | 910 | 15.6 | 0.112 | 0.7 | 42.7 |
| 148 | 0.091 | 1.07 | 0.4 | 0.2 | 0.019 | 0.394 | 16.3 | 61.6 | 22.2 | 566 | 837 | 21 | 0.128 | 1.33 | 43.5 |
| 149 | 0.054 | 2.24 | 0.14 | 0.17 | 0.02 | 0.494 | 69.5 | 4.5 | 26 | 358 | 616 | 27.8 | 0.16 | 0.89 | 73.7 |
| 150 | 0.191 | 0.85 | 0.63 | 0.19 | 0.095 | 0.515 | 29.3 | 54.8 | 15.9 | 475 | 837 | 19.6 | 0.126 | 1.06 | 37.2 |
| 151 | 0.191 | 2.1 | 0.58 | 0.14 | 0.155 | 0.717 | 15.3 | 28.6 | 56 | 739 | 1068 | 9.1 | 0.087 | 0.74 | 36 |
| 152 | 0.056 | 1.33 | 0.18 | 0.04 | 0.011 | 0.322 | 17.7 | 5.4 | 77 | 755 | 1027 | 15 | 0.1 | 1.25 | 20.3 |
| 153 | 0.07 | 1.26 | 0.2 | 0.23 | 0.166 | 0.4 | 53.5 | 7.3 | 39.2 | 542 | 780 | 17.1 | 0.128 | 0.88 | 29.8 |
| 154 | 0.174 | 1.12 | 0.6 | 0.48 | 0.056 | 0.587 | 25.1 | 4.6 | 70.3 | 630 | 1095 | 11.4 | 0.088 | 0.93 | 17.1 |
| 155 | 0.093 | 1.63 | 0.49 | 0.27 | 0.197 | 0.557 | 36.1 | 6.2 | 57.7 | 617 | 919 | 12.7 | 0.107 | 1.38 | 16.8 |
| 156 | 0.128 | 1.22 | 0.13 | 0.47 | 0.16 | 0.484 | 33.3 | 7.9 | 58.8 | 608 | 978 | 12.4 | 0.101 | 0.73 | 30.2 |
| 157 | 0.065 | 1.5 | 0.45 | 0.29 | 0.04 | 0.472 | 17.8 | 4.9 | 77.3 | 672 | 1024 | 13.3 | 0.098 | 1.25 | 18.4 |
| 158 | 0.186 | 1.9 | 0.21 | 0.06 | 0.028 | 0.561 | 47.8 | 10.1 | 42.1 | 642 | 872 | 13.5 | 0.113 | 1.04 | 30 |
| 159 | 0.092 | 1.29 | 0.12 | 0.48 | 0.021 | 0.43 | 20.1 | 62.8 | 17.1 | 478 | 741 | 21.3 | 0.138 | 0.93 | 48.7 |
| 160 | 0.166 | 1.32 | 0.44 | 0.18 | 0.169 | 0.544 | 22.3 | 1.7 | 76 | 647 | 1123 | 10.7 | 0.084 | 1.08 | 16.4 |
| 161 | 0.064 | 1.94 | 0.34 | 0.13 | 0.088 | 0.498 | 28 | 11.4 | 60.6 | 597 | 916 | 14.1 | 0.11 | 1.29 | 35.6 |
| 162 | 0.163 | 0.87 | 0.43 | 0.49 | 0.157 | 0.525 | 20.4 | 26.6 | 53 | 693 | 980 | 13.3 | 0.102 | 1.28 | 29.5 |
| 163 | 0.187 | 1.39 | 0.21 | 0.2 | 0.141 | 0.529 | 18 | 37.5 | 44.5 | 647 | 1006 | 13.6 | 0.1 | 1.04 | 26.6 |
| 164 | 0.116 | 0.86 | 0.38 | 0.01 | 0.088 | 0.355 | 22.5 | 4.9 | 72.7 | 678 | 1038 | 12.4 | 0.095 | 1.02 | 25.2 |
| 165 | 0.098 | 1.46 | 0.61 | 0.35 | 0.163 | 0.564 | 44.1 | 40.6 | 15.3 | 461 | 690 | 16.5 | 0.136 | 1.09 | 68.3 |
| 166 | 0.078 | 1.46 | 0.11 | 0.49 | 0.036 | 0.449 | 32.4 | 23.6 | 44 | 529 | 852 | 12.5 | 0.114 | 0.76 | 33.9 |
| 167 | 0.196 | 0.84 | 0.65 | 0.46 | 0.052 | 0.569 | 21.6 | 12.2 | 66.2 | 769 | 1055 | 11.5 | 0.092 | 1.39 | 24.7 |
| 168 | 0.177 | 1.32 | 0.6 | 0.18 | 0.184 | 0.591 | 53.8 | 28.9 | 17.3 | 425 | 763 | 17.1 | 0.129 | 1.1 | 30.9 |
| 169 | 0.058 | 0.88 | 0.11 | 0.31 | 0.047 | 0.297 | 25.2 | 17.3 | 57.5 | 530 | 898 | 15.6 | 0.114 | 1.18 | 39.5 |
| 170 | 0.097 | 1.05 | 0.57 | 0.11 | 0.175 | 0.443 | 30.5 | 11.5 | 58 | 671 | 963 | 13.5 | 0.104 | 0.91 | 28.3 |
| 171 | 0.193 | 2.44 | 0.28 | 0.45 | 0.163 | 0.778 | 64.6 | 10.7 | 24.8 | 544 | 825 | 20.5 | 0.128 | 1.3 | 31.5 |
| 172 | 0.146 | 1.39 | 0.42 | 0.14 | 0.116 | 0.514 | 23.3 | 21.5 | 55.2 | 679 | 976 | 15 | 0.105 | 1.12 | 34.4 |
| 173 | 0.174 | 2.47 | 0.67 | 0.08 | 0.007 | 0.739 | 71.4 | 2.2 | 26.5 | 494 | 771 | 19.2 | 0.132 | 1.26 | 30.5 |
| 174 | 0.073 | 0.85 | 0.27 | 0.24 | 0.156 | 0.347 | 15.6 | 47.2 | 37.2 | 621 | 837 | 21.7 | 0.129 | 0.94 | 42.3 |
| 175 | 0.198 | 1.23 | 0.19 | 0.03 | 0.085 | 0.465 | 23.9 | 11.5 | 64.6 | 747 | 1101 | 11.9 | 0.088 | 0.81 | 15.6 |

| | | | | | | | | | | | | | | | |
|---|---|---|---|---|---|---|---|---|---|---|---|---|---|---|---|
| 176 | 0.176 | 1.91 | 0.62 | 0.43 | 0.108 | 0.727 | 53.9 | 4.1 | 42 | 655 | 897 | 12.7 | 0.109 | 1.06 | 26.9 |
| 177 | 0.09 | 2.25 | 0.38 | 0.02 | 0.088 | 0.562 | 27.3 | 1.6 | 71.1 | 730 | 986 | 12.8 | 0.101 | 1.14 | 25.5 |
| 178 | 0.103 | 1.89 | 0.61 | 0.25 | 0.018 | 0.594 | 42.4 | 14 | 43.7 | 577 | 834 | 20 | 0.127 | 0.94 | 28.4 |
| 179 | 0.195 | 1.3 | 0.52 | 0.32 | 0.186 | 0.617 | 29.6 | 54 | 16.4 | 571 | 825 | 16.6 | 0.122 | 0.77 | 44.4 |
| 180 | 0.156 | 2.39 | 0.53 | 0.02 | 0.111 | 0.686 | 28.1 | 2.8 | 69.1 | 613 | 1041 | 14 | 0.097 | 1.06 | 18.1 |
| 181 | 0.197 | 2.31 | 0.36 | 0.38 | 0.167 | 0.764 | 35.8 | 0.4 | 63.9 | 625 | 1057 | 9.1 | 0.088 | 0.77 | 27.5 |
| 182 | 0.161 | 1.54 | 0.35 | 0.23 | 0.049 | 0.544 | 28.9 | 32.5 | 38.6 | 642 | 886 | 15.2 | 0.114 | 1.37 | 24.5 |
| 183 | 0.185 | 1.05 | 0.38 | 0.17 | 0.199 | 0.51 | 46.5 | 37.3 | 16.2 | 609 | 816 | 20.5 | 0.129 | 0.88 | 45.7 |
| 184 | 0.13 | 2.16 | 0.53 | 0.36 | 0.082 | 0.684 | 57.3 | 19.4 | 23.4 | 556 | 744 | 18.4 | 0.133 | 1.22 | 34.4 |
| 185 | 0.14 | 1.78 | 0.48 | 0.06 | 0.062 | 0.559 | 19.2 | 61.3 | 19.5 | 565 | 811 | 17.3 | 0.125 | 1.1 | 48.7 |
| 186 | 0.136 | 2.29 | 0.52 | 0.02 | 0.175 | 0.661 | 37 | 37.4 | 25.6 | 522 | 772 | 20.6 | 0.134 | 0.77 | 30.1 |
| 187 | 0.057 | 1.59 | 0.15 | 0.31 | 0.185 | 0.451 | 20.9 | 15.4 | 63.7 | 616 | 960 | 13.5 | 0.104 | 1.31 | 31.1 |
| 188 | 0.131 | 1.3 | 0.23 | 0.04 | 0.114 | 0.424 | 39.5 | 17.4 | 43.2 | 636 | 910 | 14.8 | 0.111 | 0.86 | 25.5 |
| 189 | 0.104 | 1.91 | 0.59 | 0.29 | 0.012 | 0.6 | 20.7 | 1.7 | 77.6 | 805 | 1078 | 9.4 | 0.086 | 0.89 | 26.9 |
| 190 | 0.156 | 2.51 | 0.63 | 0.33 | 0.169 | 0.8 | 29.1 | 0.9 | 70 | 659 | 1055 | 9.9 | 0.089 | 1.06 | 18.6 |
| 191 | 0.134 | 2.62 | 0.29 | 0.1 | 0.08 | 0.665 | 19.7 | 6.8 | 73.5 | 631 | 1084 | 9.3 | 0.086 | 1 | 18.3 |
| 192 | 0.054 | 1.35 | 0.4 | 0.14 | 0.071 | 0.4 | 41.1 | 5.7 | 53.2 | 596 | 844 | 16.2 | 0.12 | 1.31 | 26 |
| 193 | 0.134 | 2.43 | 0.27 | 0.29 | 0.077 | 0.668 | 25.5 | 18.4 | 56 | 563 | 999 | 12.9 | 0.099 | 0.75 | 35.3 |
| 194 | 0.177 | 2.62 | 0.26 | 0.46 | 0.187 | 0.795 | 39.9 | 41.5 | 18.6 | 487 | 771 | 16.4 | 0.127 | 0.93 | 43.1 |
| 195 | 0.141 | 0.95 | 0.37 | 0.38 | 0.091 | 0.467 | 37.8 | 2.1 | 60.2 | 544 | 949 | 13.6 | 0.106 | 0.7 | 22.1 |
| 196 | 0.18 | 1.2 | 0.28 | 0.36 | 0.028 | 0.516 | 23.2 | 9.6 | 67.2 | 788 | 1058 | 10.4 | 0.09 | 0.78 | 25.7 |
| 197 | 0.088 | 2.2 | 0.46 | 0.43 | 0.123 | 0.657 | 22.6 | 15.5 | 61.9 | 614 | 996 | 14.9 | 0.103 | 0.74 | 25 |
| 198 | 0.186 | 1.41 | 0.18 | 0.4 | 0.08 | 0.552 | 52.2 | 1.3 | 46.5 | 556 | 918 | 14.6 | 0.11 | 0.99 | 26.7 |
| 199 | 0.194 | 2.2 | 0.33 | 0.1 | 0.05 | 0.657 | 36.8 | 39.4 | 23.9 | 513 | 824 | 17.7 | 0.124 | 0.72 | 32.6 |
| 200 | 0.056 | 1.34 | 0.13 | 0.04 | 0.193 | 0.35 | 34.7 | 18.5 | 46.8 | 503 | 818 | 20.8 | 0.129 | 0.93 | 26 |
| 201 | 0.149 | 2.06 | 0.45 | 0.47 | 0.007 | 0.677 | 25.6 | 8.6 | 65.8 | 680 | 994 | 15.7 | 0.104 | 0.7 | 24.7 |
| 202 | 0.145 | 2.02 | 0.12 | 0.12 | 0.135 | 0.557 | 24.7 | 21.2 | 54 | 571 | 1000 | 14.2 | 0.101 | 1.02 | 25.2 |
| 203 | 0.097 | 1.85 | 0.49 | 0.06 | 0.094 | 0.535 | 17.2 | 4 | 78.9 | 724 | 1028 | 12.8 | 0.096 | 1.07 | 15 |
| 204 | 0.097 | 0.95 | 0.15 | 0.34 | 0.031 | 0.359 | 22.1 | 33 | 44.9 | 613 | 888 | 14.7 | 0.113 | 1.39 | 37.4 |
| 205 | 0.066 | 2.2 | 0.15 | 0.24 | 0.182 | 0.548 | 55 | 10.1 | 34.9 | 561 | 763 | 17.5 | 0.13 | 1.09 | 34.2 |
| 206 | 0.118 | 2.31 | 0.62 | 0.15 | 0.152 | 0.688 | 23.2 | 39.1 | 37.7 | 663 | 889 | 15.3 | 0.114 | 0.97 | 26.4 |
| 207 | 0.08 | 1.28 | 0.21 | 0.13 | 0.16 | 0.394 | 18.8 | 47 | 34.3 | 566 | 817 | 18.5 | 0.126 | 1.28 | 34.8 |
| 208 | 0.068 | 1.54 | 0.54 | 0.04 | 0.037 | 0.447 | 51.1 | 4 | 44.9 | 616 | 825 | 19.4 | 0.127 | 1.03 | 34 |
| 209 | 0.106 | 2.66 | 0.45 | 0.06 | 0.066 | 0.664 | 32.6 | 22.9 | 44.6 | 657 | 906 | 14.1 | 0.11 | 0.79 | 32.2 |
| 210 | 0.082 | 2.56 | 0.69 | 0.44 | 0.048 | 0.743 | 52.2 | 14.1 | 33.6 | 555 | 742 | 21.5 | 0.138 | 1.21 | 35.7 |
| 211 | 0.087 | 2.63 | 0.36 | 0.05 | 0.032 | 0.615 | 37.9 | 20.2 | 41.9 | 549 | 811 | 21.9 | 0.132 | 0.76 | 32.1 |
| 212 | 0.06 | 2.22 | 0.36 | 0.15 | 0.19 | 0.57 | 36.7 | 11.7 | 51.6 | 619 | 881 | 15.7 | 0.116 | 1.19 | 30.3 |
| 213 | 0.176 | 1.19 | 0.48 | 0.01 | 0.137 | 0.499 | 16.1 | 17.3 | 66.6 | 636 | 1066 | 9.7 | 0.088 | 0.71 | 29.9 |
| 214 | 0.111 | 1.66 | 0.23 | 0.18 | 0.026 | 0.474 | 36.2 | 16.1 | 47.7 | 592 | 870 | 14.3 | 0.114 | 1.32 | 28.5 |
| 215 | 0.144 | 2.29 | 0.16 | 0.36 | 0.115 | 0.652 | 15.7 | 13.5 | 70.8 | 764 | 1100 | 8.5 | 0.083 | 0.84 | 17.5 |
| 216 | 0.111 | 1.58 | 0.13 | 0.01 | 0.06 | 0.413 | 51.3 | 4.8 | 44 | 620 | 860 | 15.9 | 0.118 | 1.09 | 27.4 |
| 217 | 0.135 | 2.02 | 0.2 | 0.03 | 0.191 | 0.555 | 75.7 | 4.7 | 19.5 | 446 | 674 | 21.6 | 0.145 | 0.98 | 54.4 |
| 218 | 0.149 | 1.38 | 0.44 | 0.42 | 0.09 | 0.569 | 16.3 | 6.9 | 76.8 | 767 | 1090 | 8 | 0.083 | 0.86 | 20.3 |
| 219 | 0.072 | 1.07 | 0.45 | 0.09 | 0.011 | 0.362 | 53.8 | 17.9 | 28.3 | 410 | 696 | 17.8 | 0.137 | 1.27 | 54.3 |

| | | | | | | | | | | | | | | | |
|---|---|---|---|---|---|---|---|---|---|---|---|---|---|---|---|
| 220 | 0.161 | 1.3 | 0.48 | 0.23 | 0.06 | 0.532 | 27.8 | 5.6 | 66.5 | 801 | 1068 | 10.1 | 0.088 | 0.94 | 18.5 |
| 221 | 0.134 | 2.47 | 0.27 | 0.18 | 0.139 | 0.663 | 61.6 | 17.9 | 20.5 | 440 | 750 | 18.7 | 0.133 | 0.75 | 30.9 |
| 222 | 0.143 | 1.65 | 0.67 | 0.3 | 0.078 | 0.628 | 16.6 | 47.9 | 35.5 | 519 | 926 | 14.5 | 0.109 | 0.91 | 32.6 |
| 223 | 0.165 | 2.45 | 0.37 | 0.34 | 0.022 | 0.722 | 25.8 | 5.3 | 68.9 | 719 | 1098 | 8.3 | 0.083 | 1.15 | 28.1 |
| 224 | 0.102 | 1.26 | 0.6 | 0.42 | 0.147 | 0.544 | 60.5 | 1.1 | 38.4 | 536 | 748 | 21.1 | 0.137 | 1.15 | 31.3 |
| 225 | 0.097 | 2.53 | 0.64 | 0.12 | 0.162 | 0.703 | 17.6 | 11.5 | 70.9 | 738 | 1016 | 15.2 | 0.101 | 1.18 | 27.5 |
| 226 | 0.143 | 1.32 | 0.66 | 0.06 | 0.115 | 0.531 | 45.9 | 29.1 | 25 | 496 | 810 | 20.1 | 0.129 | 0.87 | 36.5 |
| 227 | 0.084 | 1.94 | 0.22 | 0.22 | 0.186 | 0.534 | 48.9 | 21.2 | 29.9 | 488 | 769 | 19.1 | 0.132 | 1.13 | 29.7 |
| 228 | 0.163 | 1.29 | 0.61 | 0.01 | 0.046 | 0.512 | 43.1 | 5.1 | 51.8 | 713 | 966 | 14.1 | 0.104 | 0.76 | 19.3 |
| 229 | 0.11 | 1.72 | 0.58 | 0.31 | 0.049 | 0.583 | 47.9 | 21 | 31.1 | 510 | 766 | 16.2 | 0.128 | 1.2 | 30 |
| 230 | 0.154 | 1.17 | 0.68 | 0.48 | 0.08 | 0.597 | 22.7 | 4.5 | 72.8 | 767 | 1080 | 8.3 | 0.084 | 0.71 | 22.1 |
| 231 | 0.098 | 1.73 | 0.27 | 0.39 | 0.071 | 0.533 | 31.8 | 13.8 | 54.5 | 643 | 940 | 14.2 | 0.107 | 0.94 | 27.4 |
| 232 | 0.167 | 2.37 | 0.38 | 0.15 | 0.111 | 0.691 | 24.7 | 57.2 | 18.1 | 486 | 813 | 18.3 | 0.126 | 0.95 | 44.3 |
| 233 | 0.145 | 0.84 | 0.39 | 0.09 | 0.075 | 0.394 | 37.3 | 46.9 | 15.9 | 541 | 797 | 21.2 | 0.132 | 1.14 | 46.5 |
| 234 | 0.095 | 2.41 | 0.15 | 0.35 | 0.058 | 0.608 | 31.1 | 24.9 | 43.9 | 515 | 847 | 21.3 | 0.127 | 0.96 | 41.1 |
| 235 | 0.152 | 1.23 | 0.17 | 0.21 | 0.031 | 0.438 | 43.7 | 17.2 | 39.1 | 631 | 891 | 12.9 | 0.11 | 1.04 | 27 |
| 236 | 0.176 | 1.05 | 0.47 | 0.23 | 0.001 | 0.492 | 23.1 | 8.4 | 68.6 | 719 | 1048 | 14.6 | 0.097 | 1.28 | 31.7 |
| 237 | 0.057 | 0.93 | 0.51 | 0.22 | 0.165 | 0.391 | 17.7 | 42.7 | 39.6 | 539 | 806 | 17.2 | 0.125 | 1 | 39.2 |
| 238 | 0.183 | 2.01 | 0.16 | 0.1 | 0.03 | 0.576 | 19.8 | 32.8 | 47.3 | 718 | 1031 | 14 | 0.098 | 1.07 | 34.1 |
| 239 | 0.133 | 1.72 | 0.55 | 0.03 | 0.096 | 0.553 | 80.3 | 3.4 | 16.3 | 426 | 641 | 29.8 | 0.161 | 1.33 | 66.2 |
| 240 | 0.057 | 2.53 | 0.47 | 0.29 | 0.008 | 0.632 | 38.2 | 10.9 | 50.9 | 453 | 811 | 20.4 | 0.13 | 0.86 | 26.9 |
| 241 | 0.068 | 2.06 | 0.69 | 0.43 | 0.165 | 0.668 | 46.9 | 22.5 | 30.6 | 416 | 715 | 21.8 | 0.141 | 0.92 | 35.3 |
| 242 | 0.08 | 1.21 | 0.62 | 0.1 | 0.052 | 0.437 | 37.5 | 9.6 | 52.9 | 622 | 896 | 14.1 | 0.112 | 1.05 | 22.5 |
| 243 | 0.063 | 1.7 | 0.61 | 0.07 | 0.117 | 0.504 | 56.3 | 16.3 | 27.4 | 476 | 699 | 19.8 | 0.14 | 0.73 | 53.9 |
| 244 | 0.081 | 1.29 | 0.51 | 0.31 | 0.065 | 0.472 | 15.5 | 11.4 | 73.1 | 638 | 1035 | 15.8 | 0.1 | 1.11 | 31.2 |
| 245 | 0.122 | 1.96 | 0.53 | 0.4 | 0.036 | 0.64 | 15.5 | 13.9 | 70.6 | 684 | 1075 | 11.7 | 0.09 | 1.22 | 28.6 |
| 246 | 0.068 | 1.49 | 0.42 | 0.45 | 0.138 | 0.52 | 42.4 | 10.3 | 47.3 | 604 | 816 | 17.8 | 0.125 | 1.15 | 36.6 |
| 247 | 0.081 | 0.8 | 0.31 | 0.17 | 0.04 | 0.319 | 18.3 | 1.9 | 79.8 | 772 | 1075 | 11.7 | 0.09 | 1.24 | 23.1 |
| 248 | 0.145 | 0.94 | 0.59 | 0.13 | 0.095 | 0.465 | 29.5 | 12.8 | 57.6 | 589 | 972 | 13 | 0.102 | 0.84 | 20.2 |
| 249 | 0.196 | 1.05 | 0.49 | 0.36 | 0.188 | 0.578 | 25.4 | 16.8 | 57.8 | 588 | 1049 | 12.8 | 0.094 | 1.22 | 22.9 |
| 250 | 0.173 | 2.4 | 0.33 | 0.13 | 0.069 | 0.677 | 20.5 | 58.4 | 21.1 | 491 | 873 | 14.7 | 0.115 | 0.73 | 32.3 |
| 251 | 0.117 | 1.26 | 0.38 | 0.49 | 0.14 | 0.529 | 16.9 | 61.9 | 21.3 | 519 | 814 | 21.6 | 0.131 | 0.9 | 52.9 |
| 252 | 0.146 | 1.48 | 0.37 | 0.23 | 0.166 | 0.547 | 17.8 | 46.8 | 35.5 | 603 | 880 | 15.4 | 0.115 | 0.82 | 35.1 |
| 253 | 0.159 | 1.52 | 0.25 | 0.08 | 0.028 | 0.482 | 21.6 | 27.6 | 50.8 | 637 | 993 | 12.8 | 0.1 | 1.19 | 35.3 |
| 254 | 0.154 | 0.91 | 0.55 | 0.27 | 0.1 | 0.49 | 56.9 | 0.2 | 42.9 | 460 | 822 | 18.5 | 0.126 | 1.16 | 22.3 |
| 255 | 0.098 | 2.04 | 0.52 | 0.1 | 0.125 | 0.588 | 75.7 | 4.5 | 19.8 | 394 | 653 | 19.9 | 0.145 | 0.82 | 66.8 |
| 256 | 0.058 | 2.61 | 0.25 | 0.42 | 0.078 | 0.643 | 19.3 | 42 | 38.7 | 580 | 806 | 20.1 | 0.129 | 0.81 | 43.3 |
| 257 | 0.153 | 2.38 | 0.5 | 0.09 | 0.124 | 0.692 | 17.1 | 12.6 | 70.3 | 794 | 1059 | 9.6 | 0.088 | 0.71 | 26.9 |
| 258 | 0.092 | 2.6 | 0.31 | 0.29 | 0.038 | 0.653 | 29.4 | 10.3 | 60.3 | 523 | 940 | 12.1 | 0.104 | 0.78 | 33.6 |
| 259 | 0.182 | 1.12 | 0.4 | 0.38 | 0.072 | 0.541 | 24.8 | 0.3 | 75 | 728 | 1139 | 9.3 | 0.08 | 0.83 | 15 |
| 260 | 0.09 | 2.39 | 0.22 | 0.3 | 0.028 | 0.598 | 51.1 | 8.3 | 40.5 | 593 | 805 | 16.9 | 0.125 | 1.38 | 30.7 |
| 261 | 0.155 | 2.58 | 0.58 | 0.19 | 0.116 | 0.763 | 21.5 | 24.5 | 53.9 | 618 | 965 | 14.1 | 0.105 | 1.14 | 30.9 |
| 262 | 0.175 | 2.64 | 0.17 | 0.24 | 0.069 | 0.711 | 61.6 | 2 | 36.4 | 495 | 824 | 21.2 | 0.129 | 1 | 30.1 |
| 263 | 0.155 | 0.9 | 0.15 | 0.09 | 0.133 | 0.379 | 28.6 | 13.6 | 57.8 | 618 | 971 | 15.6 | 0.106 | 0.72 | 29.8 |

| | | | | | | | | | | | | | | | |
|---|---|---|---|---|---|---|---|---|---|---|---|---|---|---|---|
| 264 | 0.187 | 1.16 | 0.62 | 0.3 | 0.01 | 0.566 | 47.2 | 23.8 | 29 | 573 | 868 | 12 | 0.111 | 0.87 | 24.6 |
| 265 | 0.096 | 1.85 | 0.37 | 0.14 | 0 | 0.507 | 24.7 | 17 | 58.4 | 622 | 966 | 12.3 | 0.102 | 0.95 | 24.8 |
| 266 | 0.144 | 1.88 | 0.4 | 0.03 | 0.04 | 0.551 | 20 | 8.9 | 71.1 | 802 | 1103 | 9.2 | 0.083 | 1.21 | 15 |
| 267 | 0.159 | 2.16 | 0.23 | 0.04 | 0.079 | 0.588 | 16.1 | 9.1 | 74.9 | 732 | 1093 | 10.3 | 0.086 | 1.07 | 17.7 |
| 268 | 0.146 | 2.37 | 0.42 | 0.19 | 0.037 | 0.67 | 29 | 18.4 | 52.6 | 662 | 935 | 13.4 | 0.107 | 1.09 | 21.4 |
| 269 | 0.157 | 2.35 | 0.51 | 0.48 | 0.112 | 0.77 | 25.2 | 1.6 | 73.2 | 648 | 1076 | 9.9 | 0.087 | 1.36 | 20.5 |
| 270 | 0.175 | 1.54 | 0.54 | 0.23 | 0.178 | 0.619 | 51.9 | 9.2 | 38.9 | 522 | 896 | 15.1 | 0.113 | 0.73 | 22.5 |
| 271 | 0.198 | 0.81 | 0.26 | 0.29 | 0.035 | 0.45 | 25 | 16.5 | 58.5 | 664 | 1028 | 15.4 | 0.1 | 1.28 | 29.6 |
| 272 | 0.06 | 1 | 0.24 | 0.12 | 0.107 | 0.32 | 36.8 | 9.6 | 53.6 | 577 | 888 | 14.4 | 0.113 | 0.92 | 31.2 |
| 273 | 0.183 | 0.94 | 0.33 | 0.26 | 0.161 | 0.49 | 23.5 | 8.8 | 67.7 | 676 | 1057 | 9.1 | 0.088 | 0.72 | 27 |
| 274 | 0.171 | 1.99 | 0.37 | 0.29 | 0.109 | 0.656 | 29.9 | 0.2 | 69.9 | 677 | 1101 | 9.7 | 0.084 | 0.74 | 17.9 |
| 275 | 0.096 | 1.53 | 0.31 | 0.22 | 0.065 | 0.469 | 23.7 | 20.7 | 55.7 | 528 | 943 | 14.4 | 0.107 | 0.9 | 35.4 |
| 276 | 0.129 | 1.29 | 0.61 | 0.06 | 0.134 | 0.503 | 31.2 | 48.7 | 20 | 564 | 801 | 20.3 | 0.13 | 0.83 | 42.2 |
| 277 | 0.114 | 2.32 | 0.68 | 0.2 | 0.011 | 0.679 | 73.9 | 0.3 | 25.8 | 444 | 722 | 19.9 | 0.138 | 0.93 | 28.3 |
| 278 | 0.128 | 2.51 | 0.55 | 0.43 | 0.017 | 0.745 | 19.1 | 20.7 | 60.2 | 615 | 973 | 13 | 0.102 | 0.85 | 28.2 |
| 279 | 0.092 | 2.36 | 0.36 | 0.32 | 0.099 | 0.639 | 23.7 | 1.2 | 75.1 | 764 | 1027 | 14 | 0.098 | 1.15 | 28.9 |
| 280 | 0.096 | 2.1 | 0.41 | 0.34 | 0.154 | 0.627 | 37.6 | 18.2 | 44.2 | 554 | 839 | 20.8 | 0.127 | 0.8 | 32.1 |
| 281 | 0.19 | 2.58 | 0.37 | 0.32 | 0.075 | 0.773 | 20.8 | 0.6 | 78.6 | 851 | 1144 | 10.9 | 0.082 | 1.23 | 15 |
| 282 | 0.167 | 2.49 | 0.3 | 0.13 | 0.002 | 0.668 | 37.5 | 31.3 | 31.2 | 604 | 884 | 15 | 0.114 | 1.38 | 28.9 |
| 283 | 0.148 | 0.82 | 0.51 | 0.28 | 0.038 | 0.451 | 35.1 | 47.6 | 17.4 | 517 | 768 | 20 | 0.133 | 1.08 | 47.4 |
| 284 | 0.11 | 1.06 | 0.31 | 0.48 | 0.01 | 0.444 | 25.5 | 21.5 | 53.1 | 629 | 950 | 14.3 | 0.106 | 0.97 | 25.1 |
| 285 | 0.184 | 1.93 | 0.16 | 0.37 | 0.038 | 0.619 | 35 | 8.9 | 56.1 | 623 | 1034 | 12.6 | 0.095 | 1.21 | 23.8 |
| 286 | 0.081 | 2.39 | 0.69 | 0.18 | 0.142 | 0.68 | 17.5 | 3 | 79.5 | 627 | 1016 | 15.1 | 0.101 | 0.84 | 22 |
| 287 | 0.168 | 1.11 | 0.23 | 0.42 | 0.16 | 0.517 | 16.1 | 24 | 59.9 | 643 | 1041 | 12.5 | 0.095 | 0.85 | 33 |
| 288 | 0.193 | 1.8 | 0.59 | 0.04 | 0.039 | 0.628 | 31.5 | 31.4 | 37.2 | 661 | 893 | 12.5 | 0.109 | 1.28 | 29 |
| 289 | 0.181 | 1.12 | 0.69 | 0.36 | 0.02 | 0.581 | 20.9 | 15.1 | 64 | 753 | 1102 | 8.3 | 0.082 | 1.31 | 31.8 |
| 290 | 0.181 | 1.47 | 0.36 | 0.25 | 0.081 | 0.564 | 43.4 | 18.1 | 38.5 | 589 | 873 | 15.8 | 0.116 | 1.08 | 35.5 |
| 291 | 0.051 | 1.53 | 0.49 | 0.34 | 0.074 | 0.488 | 16 | 26.8 | 57.2 | 522 | 906 | 12.2 | 0.108 | 1.11 | 28.7 |
| 292 | 0.052 | 2.4 | 0.54 | 0.27 | 0.067 | 0.626 | 18.6 | 21.5 | 59.9 | 609 | 959 | 14.1 | 0.105 | 1.1 | 26.3 |
| 293 | 0.056 | 1.11 | 0.55 | 0.34 | 0.107 | 0.439 | 37.7 | 10.7 | 51.6 | 519 | 874 | 14.9 | 0.115 | 0.76 | 31.8 |
| 294 | 0.147 | 1.71 | 0.22 | 0.05 | 0.032 | 0.493 | 35.2 | 0.6 | 64.2 | 733 | 1034 | 14.2 | 0.098 | 1.2 | 17.2 |
| 295 | 0.111 | 2.13 | 0.36 | 0.18 | 0.039 | 0.58 | 65.8 | 2.8 | 31.3 | 553 | 758 | 21.2 | 0.136 | 1.05 | 37.3 |
| 296 | 0.119 | 0.8 | 0.19 | 0.13 | 0.007 | 0.318 | 57.8 | 1.4 | 40.8 | 495 | 826 | 16.5 | 0.122 | 1.12 | 25 |
| 297 | 0.173 | 0.87 | 0.4 | 0.04 | 0.161 | 0.437 | 15.9 | 16.1 | 68 | 625 | 1071 | 12 | 0.091 | 0.96 | 19.3 |
| 298 | 0.094 | 1.32 | 0.37 | 0.35 | 0.111 | 0.481 | 15.9 | 51.2 | 32.9 | 519 | 813 | 16.4 | 0.123 | 1.32 | 46.8 |
| 299 | 0.173 | 1.23 | 0.14 | 0.04 | 0.146 | 0.443 | 37.3 | 21.8 | 40.9 | 698 | 944 | 12.2 | 0.104 | 0.91 | 32 |
| 300 | 0.194 | 2.26 | 0.66 | 0.35 | 0.083 | 0.789 | 16.4 | 31.2 | 52.4 | 741 | 1045 | 13.3 | 0.095 | 1.32 | 34.2 |
